# SUMOylated NKAP is essential for chromosome alignment by anchoring CENP-E to kinetochores

**DOI:** 10.1038/ncomms12969

**Published:** 2016-10-03

**Authors:** Teng Li, Liang Chen, Juanxian Cheng, Jiang Dai, Yijiao Huang, Jian Zhang, Zhaoshan Liu, Ang Li, Na Li, Hongxia Wang, Xiaomin Yin, Kun He, Ming Yu, Tao Zhou, Xuemin Zhang, Qing Xia

**Affiliations:** 1 State Key Laboratory of Proteomics, Institute of Basic Medical Sciences, National Center of Biomedical Analysis, 27 Tai-Ping Road, Beijing 100850, China; 2 Department of Gynecology, Affiliated Hospital of Academy of Military Medical Sciences, Beijing 100071, China

## Abstract

Chromosome alignment is required for accurate chromosome segregation. Chromosome misalignment can result in genomic instability and tumorigenesis. Here, we show that NF-κB activating protein (NKAP) is critical for chromosome alignment through anchoring CENP-E to kinetochores. NKAP knockdown causes chromosome misalignment and prometaphase arrest in human cells. NKAP dynamically localizes to kinetochores, and is required for CENP-E kinetochore localization. NKAP is SUMOylated predominantly in mitosis and the SUMOylation is needed for NKAP to bind CENP-E. A SUMOylation-deficient mutant of NKAP cannot support the localization of CENP-E on kinetochores or proper chromosome alignment. Moreover, Bub3 recruits NKAP to stabilize the binding of CENP-E to BubR1 at kinetochores. Importantly, loss of NKAP expression causes aneuploidy in cultured cells, and is observed in human soft tissue sarcomas. These findings indicate that NKAP is a novel and key regulator of mitosis, and its dysregulation might contribute to tumorigenesis by causing chromosomal instability.

## Abstract (web summary)

The kinetochore-bound motor CENP-E plays a critical role in chromosome alignment. Here, the authors show that NF-κB activating protein (NKAP) dynamically localises to kinetochores, is SUMOylated during mitosis, and this modification is required for NKAP to bind CENP-E and localise CENP-E to the kinetochore.

## Graphical abstract

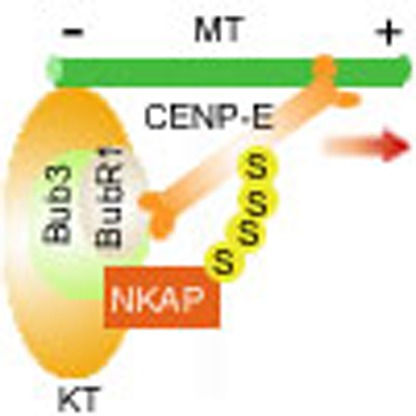

Proper chromosome alignment is critical for accurate chromosome segregation in mitosis^1^. To facilitate the successful chromosome alignment, kinetochores need to be attached by microtubules properly^2^. Composed of multiple protein complexes, the kinetochore is structured in a highly hierarchical fashion and undergoes a dynamic assembly process on entry into mitosis^3^. Whereas some core components, such as the constitutive centromere-associated network (CCAN) proteins CENP-A and CENP-C, localize to the inner kinetochore throughout the cell cycle^4^, many other proteins localize to the outer kinetochore transiently during mitosis^5^,^6^. These proteins include kinetochore-bound motor proteins CENP-E and dynein, as well as the spindle assembly checkpoint (SAC) proteins, such as Bub3, BubR1 and Mad2 (refs 5, 7). The correct localization and function of these kinetochore proteins are essential for proper chromosome alignment and faithful chromosome segregation^8^.

Accumulating evidences have demonstrated that the kinetochore-bound motor CENP-E plays critical roles in chromosome alignment^9^,^10^. CENP-E is composed of an N-terminal motor domain, a coiled-coil domain and a C-terminal tail domain. The tail domain (aa 1958–2701) is believed to be sufficient for CENP-E targeting to kinetochores^11^. CENP-E is dynamically located on the outer kinetochore from prometaphase to anaphase, and plays critical roles in the stabilization of kinetochore–microtubule (KT–MT) attachment and congression of polar-localized chromosomes to the metaphase plate^12^,^13^. When CENP-E is knocked down, a fraction of chromosomes fail to congress to the spindle equator but lie near the spindle poles^14^,^15^. For those CENP-E-free chromosomes aligned along the spindle equator, the number of the microtubules attached to the kinetochores is significantly decreased^16^,^17^,^18^,^19^. The proper kinetochore localization of CENP-E is critical for its function in mitosis. Several proteins have been reported to regulate CENP-E kinetochore localization, such as BubR1, Bub3, Bub1, CENP-F and Mad1 (refs 20, 21, 22, 23, 24).

NF-κB activating protein (NKAP) is initially reported as a possible regulator of NF-κB activation^25^. Recent studies have shown that NKAP is a RNA-binding protein and involves in T cell development^26^,^27^. Through a mitotic regulator screening, we found that NKAP depletion resulted in significant mitotic arrest. In this study, we demonstrate that NKAP is a novel mitotic regulator that plays a key role in chromosome alignment. NKAP knockdown results in the failure of CENP-E localization on kinetochores and consequently leads to KT–MT attachment defect and chromosome misalignment. NKAP undergoes SUMOylation in mitosis and SUMOylated NKAP is required for the recruitment of CENP-E to kinetochores. In addition, loss of NKAP causes chromosome missegregation and aneuploidy and is observed in human soft tissue sarcomas.

## Results

### NKAP knockdown causes chromosome misalignment

Several large-scale screening studies have been carried out to identify cell cycle-associated genes^28^,^29^,^30^. Neumann *et al*.^28^ performed a genome-wide cell division phenotype profiling ( http://www.mitocheck.org). Matthias Mann's group^29^ and M.W. Kirschner's group^30^ applied mass spectrometry-based proteomics strategy to investigate the global posttranslational modifications (PTM) during cell-cycle progression, such as phosphorylation, ubiquitination and SUMOylation. To identify novel mitotic regulators, we selected 518 genes from these screening databases as candidates (Supplementary Fig. 1a). All of the candidate genes are implied, but have never been proven, to be involved in cell-cycle regulation previously. Using several known mitotic regulators, such as P31, UBCH10 as positive controls, we carried out a siRNA-based screening of these candidate genes using mitotic index as readout (Supplementary Fig. 1b). The screening result was provided in Supplementary Data 1. As listed in Supplementary Fig. 1c, NKAP is one of the candidates with high mitotic index (35.33%).

**Figure 1:**
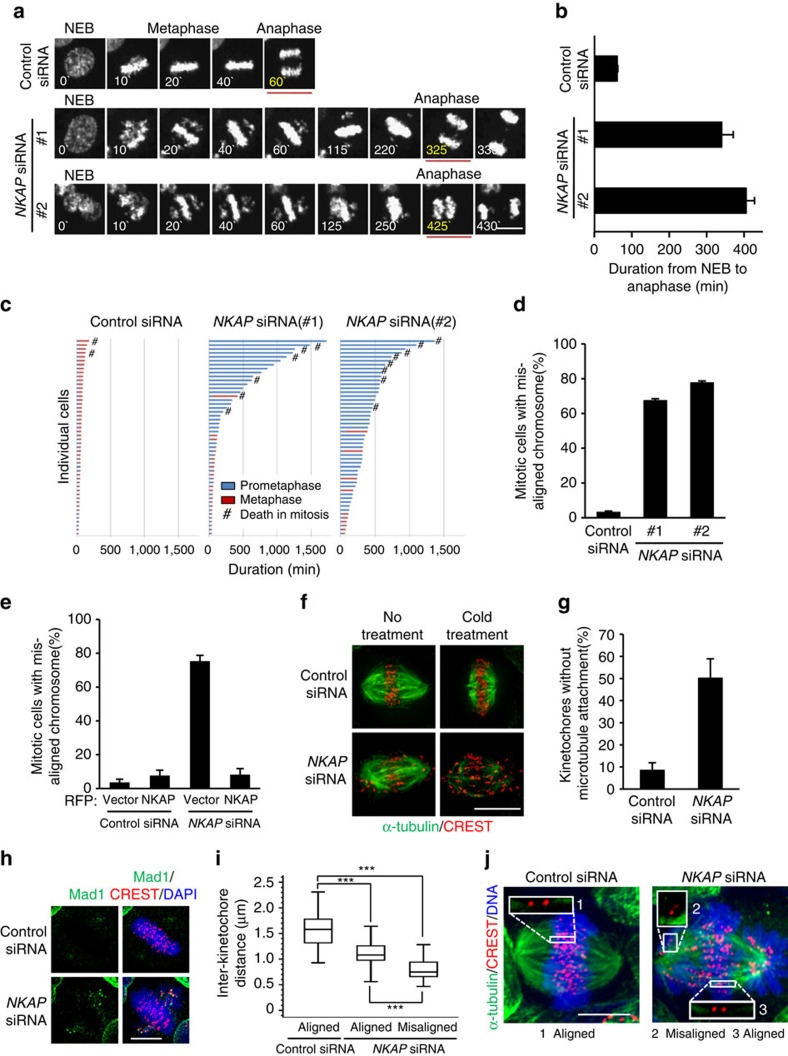
NKAP is required for chromosome alignment and KT–MT attachment. (**a**) Selected frames from time-lapse movies of representative HeLa/GFP-H2B cells transfected with the indicated siRNA. The time on the images is in minutes. NEB, nuclear envelope breakdown. See Supplementary Movies 1-3. (**b**) The lengths of time from NEB to anaphase onset in control cells (*n*=185), *NKAP* siRNA #1 cells (*n*=219) and #2 cells (*n*=188) as described in **a** were analysed. Data are mean±s.e.m. (**c**) Graph of the cumulative duration of mitotic phases in cells as described in **a**. Each bar represents one cell. (**d**) Percentage of mitotic cells with misaligned chromosomes in control or NKAP-knockdown cells. Data are mean±s.d. (**e**) Complementation of RFP-NKAP in NKAP-knockdown cells rescues chromosome misalignment. The control cells were transfected with RFP vector (*n*=234) or *NKAP* siRNA #2-resistant RFP-NKAP (*n*=220), and the NKAP-knockdown cells were transfected with RFP vector (*n*=230) or *NKAP* siRNA #2-resistant RFP-NKAP (*n*=243). Data are mean±s.d. (**f**) Instability of KT–MT attachment in NKAP-depleted cells. The indicated siRNA-transfected HeLa cells were treated with MG132 for 2 h and then incubated on ice for 5 min or not before fixation. Cells were stained for kinetochores (CREST, red) and microtubules (α-tubulin, green). (**g**) Percentage of kinetochores with complete lack of microtubule attachment from cells as described in **f** (*n*>500 kinetochores for each group of each experiment). Data are mean±s.d. Cells were randomly selected for observation and statistics. (**h**) Mad1 was activated on misaligned kinetochores in NKAP-depleted cells. Cells were stained for kinetochores (CREST, red), Mad1 (green) and DNA (blue). (**i**) Reduced inter-kinetochore distance in NKAP-knockdown cells. Average inter-kinetochore distances were quantified by measuring the distance between sister chromatids. Approximately 100 kinetochore pairs from 10 to 15 cells were measured for each experiment). ***P<0.001 (One-way ANOVA). (**j**) Representative images for cells as described in **i**. Insets (1–3) show individual inter-kinetochore distance from aligned or misaligned chromosomes. Every experiment was reproduced three times independently. All scale bars indicate 10 μm.

To further verify the function of NKAP in mitosis, we monitored the mitotic progression by time-lapse imaging of HeLa/GFP-H2B cells. We found that NKAP knockdown caused marked delay of anaphase onset (Fig. 1a, Supplementary Fig. 2a and Supplementary Movies 1–3). The average duration increased from 60.86±1.37 min (mean±s.e.m.) in control cells to 340.7±30.8 min in *NKAP* siRNA #1 treated cells and 406.1±21.9 min in siRNA #2 treated cells (Fig. 1b). We further found that NKAP-knockdown-induced delay of anaphase initiation was resulted from a significant arrest of prometaphase (Fig. 1c). Moreover, we observed that NKAP depletion resulted in significant increases of mitotic cells with misaligned chromosomes (Fig. 1d). NKAP-knockdown-induced mitotic arrest was also confirmed in other three cell lines (Supplementary Fig. 2b–d). In addition, we performed rescue experiments with siRNA #2-resistant NKAP, and found that NKAP-knockdown-induced chromosome misalignment and prometaphase arrest could be effectively reversed (Fig. 1e and Supplementary Fig. 2e,f). The *NKAP* siRNA #2 was used in the following experiments unless indicated otherwise. We further demonstrated that the prometaphase arrest was a SAC-dependent event, since the depletion of key SAC protein BubR1 or Mad2 overrode the NKAP-knockdown-induced mitotic arrest (Supplementary Fig. 2g). We also excluded the defects before mitotic entry in NKAP-depleted cells (Supplementary Fig. 2h). Collectively, these results suggest that NKAP plays a key role in chromosome alignment.

### NKAP knockdown impairs KT–MT attachment

Since chromosome misalignment is frequently resulted from KT–MT attachment defects, we then investigated whether KT–MT attachment was impaired in NKAP-knockdown cells. We first determined the stability of kinetochore fibres (K-fibres) by using cold treatment to depolymerize unstable microtubules. The results showed that the majority of kinetochores were scattered without microtubules attached in NKAP-depleted cells, whereas the kinetochores in control cells remained on metaphase plate with microtubules attached properly (Fig. 1f,g). We also checked the status of Mad1 on kinetochores, which has been known to specifically localize on unattached kinetochores^7^,^31^. The result showed that Mad1-positive kinetochores were absent in most control cells, while over 80% NKAP-depleted cells displayed more than two kinetochores positive for Mad1 (Fig. 1h; Supplementary Fig. 2i). This result further confirmed that depletion of NKAP impaired KT–MT attachment. Moreover, the inter-kinetochore distances were examined in NKAP-knockdown cells. We found that NKAP knockdown caused a significant reduction of inter-kinetochore distances in both aligned and misaligned chromosomes, indicating that NKAP depletion decreased the tension between sister kinetochores (Fig. 1i,j). Since the failure of K-fibre maturation is another reason for chromosome misalignment^32^, we also performed microtubule regrowth assay and the result excluded this possibility (Supplementary Fig. 2j). Taken together, these results demonstrate that the defect of KT–MT attachment results in chromosome misalignment in NKAP-depleted cells.

### NKAP knockdown impairs CENP-E KT localization

**Figure 2:**
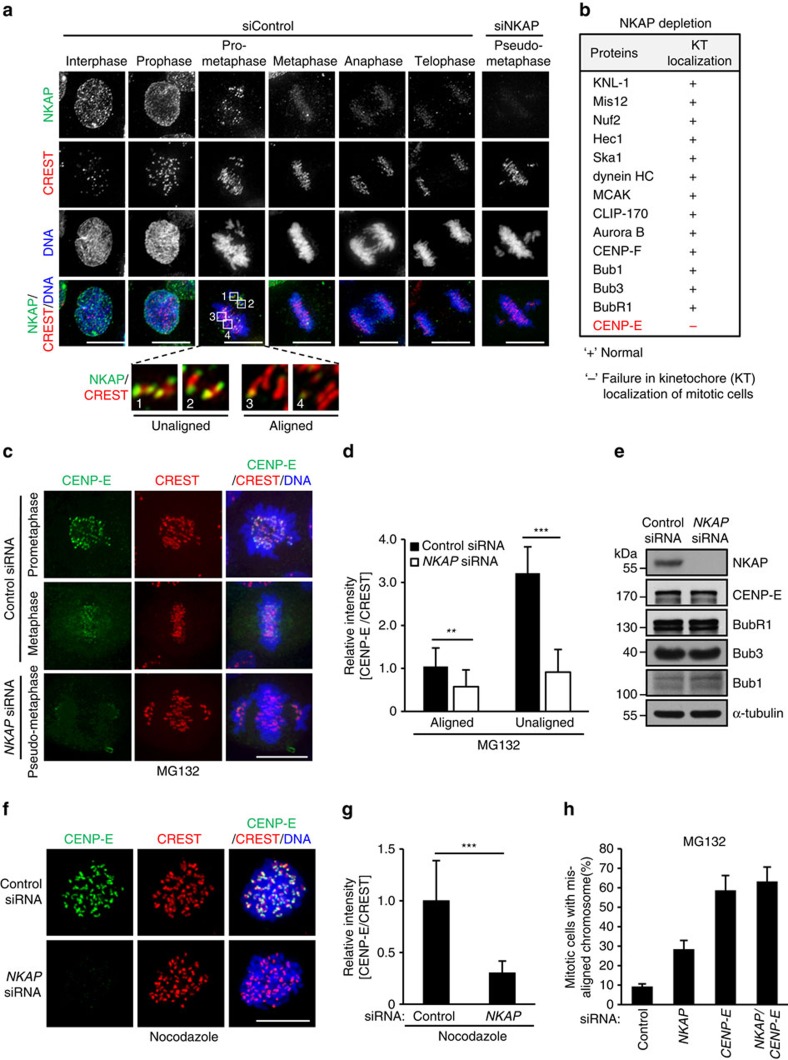
NKAP depletion impairs CENP-E kinetochore localization. (**a**) Immunofluorescence staining of NKAP in HeLa cells transfected with control or *NKAP* siRNA. The boxed regions are magnified to show typical NKAP localization on kinetochores during prometaphase. (**b**) Table summarizing the effect of NKAP depletion on kinetochore/centromere localization of the indicated proteins. HeLa cells were transfected with siRNAs for 48 h before MG132 treatment for 2 h. ‘+' and ‘−' represent normal or failure in kinetochores localization of the proteins in NKAP-knockdown cells, respectively. (**c**) Decreased CENP-E localization on kinetochores in NKAP-depleted cells. HeLa cells were treated as described in **b** and then stained for CENP-E (green), CREST (red) and DNA (blue). (**d**) Quantification of relative fluorescence intensity of the CENP-E signal at kinetochores on aligned or misaligned chromosomes. Unaligned chromosomes of the control cells were selected from prometaphase cells. Fluorescence intensity of CENP-E on aligned kinetochores in control cells were normalized to 1. Data are shown as mean±s.d. *n*≥200 kinetochores from 20 cells for each group of each experiment. **P<0.01, ***P<0.001 (two-sided student's *t*-test). (**e**) HeLa cells were treated as described in **b**, and analysed by immunoblots with the indicated antibodies. (**f**) Decreased CENP-E localization on kinetochores in NKAP-depleted cells at prometaphase. HeLa cells were treated with control or *NKAP* siRNA for 48 h followed by arresting with nocodazole. Cells were stained for CENP-E (green), CREST (red) and DNA (blue). (**g**) Quantification of relative fluorescence intensity of the CENP-E signal on kinetochores in cells as described in **f**. Fluorescence intensity of CENP-E in control cells was normalized to 1 (*n*≥5 cells, >200 kinetochores of each experiment). Data are shown as mean±s.d. and are representative of three independent experiments. ***P<0.001 (two-sided Mann–Whitney *U* test). (**h**) Quantitative analysis of mitotic cells with misaligned chromosomes. HeLa cells were transfected with the indicated siRNAs for 48 h, and then treated with MG132 for 2 h. Cells were randomly selected for observation and statistics. Data are representative of three independent experiments and shown as mean±s.d. All scale bars indicate 10 μm.

To further study how NKAP regulates KT–MT attachment, we first investigated the cellular localization of endogenous NKAP during mitosis. To do so, we prepared the monoclonal antibody against NKAP (mAb A2-5) for immunostaining. The antibody specificity of mAb A2-5 was validated by the disappearance of signals in NKAP-depleted cells in immunostaining and western blot analyses (Supplementary Fig. 3a,b). The immunostaining results showed that NKAP was mainly distributed in nuclei of interphase and prophase cells. In prometaphase, NKAP protein was found to be mainly localized at kinetochores (Fig. 2a). Notably, we observed NKAP proteins concentrated at kinetochores of unaligned chromosomes, whereas significantly diminished at aligned kinetochores (magnified boxed regions 1–4 in Fig. 2a). In addition, by isolating mitotic chromosome, we demonstrated that NKAP exclusively localized in chromosome fraction but not supernatant (Supplementary Fig. 3c). These results indicate that NKAP mainly localizes on chromosomes including kinetochores in mitosis, suggesting it plays a role in chromosome alignment during prometaphase.

To further analyze the molecular mechanism how NKAP regulates KT–MT attachment, we investigated whether NKAP depletion affected the kinetochore/centromere localization of the proteins, which have been known to play critical roles in KT–MT attachment. Among the candidates listed in Fig. 2b, we found that CENP-E was the only protein whose localization on kinetochore was obviously affected in NKAP-knockdown cells (Fig. 2b; Supplementary Fig. 3d–k). The CENP-E signal intensity on kinetochores was significantly decreased for both aligned and misaligned chromosomes in NKAP-knockdown cells (Fig. 2c,d). Since previous reports suggested that NKAP is a transcriptional corepressor^26^, we performed a real-time PCR analysis and the result showed that NKAP knockdown did not affect the transcription of CENP-E (Supplementary Fig. 3l). We also demonstrated that NKAP knockdown had no effect on the protein level of CENP-E and other proteins, which have been reported to regulate CENP-E kinetochore localization (Fig. 2e). Moreover, similar with NKAP, CENP-E knockdown resulted in a marked increase of the depolymerization of microtubules on cold treatment, which is consistent with the effect of NKAP knockdown on CENP-E kinetochore localization (Supplementary Fig. 3m).

Since CENP-E is known to be initially recruited to kinetochores in prometaphase, we next investigated whether the recruitment of CENP-E to kinetochore was affected by NKAP depletion. To do so, we arrested the cells in prometaphase with nocodazole treatment, and performed the immunostaining assay. The result showed that CENP-E immunofluorescence signal on kinetochores was markedly decreased in NKAP-depleted cells (Fig. 2f,g). We also examined the effect of CENP-E knockdown on NKAP localization, and no obvious change of NKAP localization was observed in CENP-E-depleted cells (Supplementary Fig. 3n). These results suggest that NKAP is required for the recruitment of CENP-E to kinetochores in prometaphase.

To further investigate whether CENP-E is involved in NKAP depletion-induced chromosome misalignment, we examined the effect of NKAP or/and CENP-E knockdown on chromosome alignment. The knockdown efficiency is verified in all the samples by western blot (Supplementary Fig. 3o). As indicated in Fig. 2h, depletion of either NKAP or CENP-E resulted in marked increase of cells with chromosome misalignment. However, when NKAP and CENP-E were depleted together, no additional effect on chromosome misalignment was observed compared with CENP-E depletion, suggesting that NKAP plays a role in chromosome alignment through regulating CENP-E. We noticed that CENP-E knockdown led to more severe phenotype than NKAP knockdown did, which is likely due to the possibility that other previously reported proteins such as Mad1 (ref. 24), TRAMM/TrappC12 (ref. 33) and Nuf2 (ref. 34) also contribute to CENP-E kinetochore localization.

### NKAP is SUMOylated in mitosis

**Figure 3:**
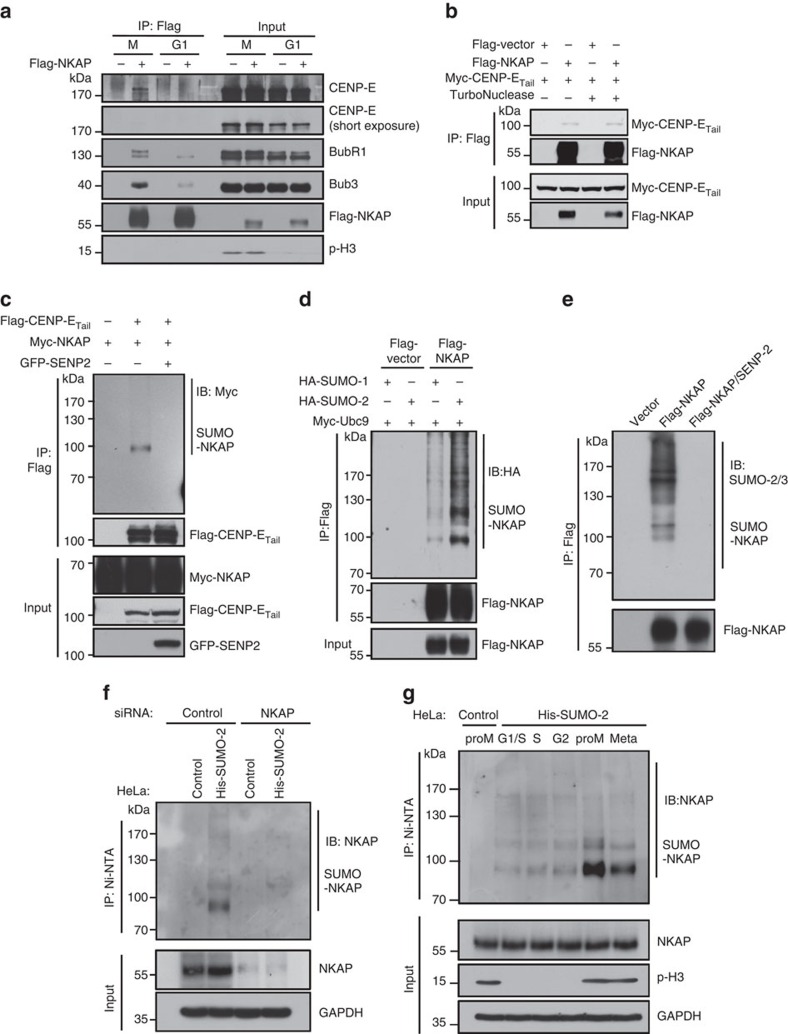
NKAP undergoes SUMOylation and interacts with CENP-E in mitosis. (**a**) NKAP is associated with kinetochore proteins including CENP-E. HeLa cells were transfected with Flag-tagged vector or NKAP and synchronized at the indicated cell-cycle phases. Cell lysates were immunoprecipitated with anti-Flag M2 beads and the IPs were analysed by immunoblot with indicated antibodies. Phosphorylated histone H3 (Ser-10) acts as an indicator of M phase. (**b**) A co-IP experiment detecting the interaction between NKAP and CENP-E. HEK293T cells were co-transfected with the indicated tagged expression vectors and then treated with nocodazole for 16 h. The cell lysates with or without TurboNuclease were immunoprecipitated with anti-Flag M2 beads and analysed by immunoblot using indicated antibodies. (**c**) A reversed co-IP experiment detecting the interaction between NKAP and CENP-E. HEK293T cells were co-transfected with vectors expressing Flag-CENP-E_Tail_ and Myc-NKAP, with or without GFP-SENP2, and then immunoprecipitated with anti-Flag M2 beads and analysed by immunoblot. (**d**) *In vivo* SUMOylation assay of NKAP. HEK293T cells were transiently transfected with vectors expressing Flag-NKAP, HA-SUMO-1 or -2 together with the E2 SUMO ligase Ubc9, as indicated. After 48 h of transfection, cells were lysed and immunoprecipitated. Flag-tagged IPs were immunoblotted with the indicated antibodies. (**e**) *In vivo* SUMOylation assay of NKAP without overexpressing Ubc9 or SUMO-2 in HEK293T cells. (**f**) Detecting the SUMOylation of endogenous NKAP in mitotic HeLa cells. HeLa/His-SUMO-2 and parental cells were transfected with control or *NKAP* siRNA as indicated. His-SUMO-2 conjugates were recovered on Ni-NTA beads and analysed by immunoblot with indicated antibodies. (**g**) HeLa/His-SUMO-2 and parental cells were synchronized at the indicated cell-cycle phases. His-SUMO-2 conjugates were recovered and analysed by immunoblot with indicated antibodies. proM, prometaphase; Meta, metaphase.

To explore how NKAP promotes CENP-E localization on kinetochores, we performed immunoprecipitation (IP) experiment to examine whether NKAP was associated with CENP-E. HeLa cells were synchronized in mitosis and G1 phase, respectively. As shown in Fig. 3a, CENP-E as well as BubR1 and Bub3 were found in the Flag-NKAP immunocomplexes in mitotic cells, but much less in G1 cells. Since NKAP is a RNA-binding protein^27^, we added TurboNuclease into cell lysis for IP experiment, and the results excluded the possibility that the association of NKAP with CENP-E was mediated by RNAs (Fig. 3b). We also performed the gel filtration experiment. The result clearly showed NKAP, CENP-E, BubR1 and Bub3 partially co-fractionated (Supplementary Fig. 4a), consistent with their association in IP experiment. In a separate experiment to identify NKAP interacting proteins, we performed affinity purification using lysates of prometaphase HeLa cells that stably expressed Flag-vector/Flag-NKAP. Through mass spectrometry analysis, we identified several kinetochore proteins, such as Bub3, BubR1 and Bub1, in the Flag-NKAP immunocomplex (Supplementary Fig. 4b–d). These data support that NKAP is associated with CENP-E in the kinetochore protein complex.

We further performed a reverse co-immunoprecipitating (co-IP) experiment using Flag-CENP-E_Tail_ as a bait. Unexpectedly, the anti-Myc antibody detected no band at ∼ 60 kDa, the theoretical molecular weight of Myc-NKAP, while smeared bands over 90 kDa were detected in the IPs (Supplementary Fig. 4e). Since several recent large-scale analyses by mass spectrometry identified NKAP as a SUMO-2/3 substrate^35^, we performed the same co-IP experiment as shown in Supplementary Fig. 4e in the presence of GFP-SENP2, a deSUMOylating enzyme. Indeed, the smeared bands over 90 kDa disappeared as a result of GFP-SENP2 expression (Fig. 3c). These results suggest that these bands are most likely to be the SUMOylated forms of NKAP, and CENP-E interacts with the SUMOylated NKAP.

To verify the SUMOylation of NKAP, HEK293T cells were transfected with Flag-NKAP, SUMO-E2 conjugating enzyme Ubc9 and the indicated HA-tagged SUMO isoforms. Using Flag-NKAP as a bait in the IP experiment, we demonstrated that NKAP was obviously SUMOylated, mainly in the form of SUMO-2 (Fig. 3d). With HeLa cells stably expressing His-SUMO-2 proteins, we further confirmed the SUMOylation of NKAP using His-SUMO-2 as a bait in the IP experiment, and the smeared bands were no longer existed in the presence of SENP2 (Supplementary Fig. 4f). We also detected NKAP SUMOylation *in vivo* without overexpressing Ubc9 and SUMO-2 in HEK293T cells. As shown in Fig. 3e, anti-SUMO-2/3 antibody detected smeared bands over 90 kDa in the Flag-NKAP IPs, which disappeared in the SENP2 co-expressed cells. To detect the endogenous SUMOylation of NKAP, we performed denaturing purification of His-SUMO conjugates using HeLa/His-SUMO-2 stable cell line and blotted the IPs with antibody of NKAP. As shown in Fig. 3f, the SUMOylated NKAP was detected in the control cells but not in the NKAP-knockdown cells with anti-NKAP antibody. This data indicates that the endogenous NKAP is SUMOylated in prometaphase at least when SUMO is ectopically expressed. We further examined the kinetics of NKAP SUMOylation during the cell-cycle process. The results demonstrated that NKAP underwent the strongest SUMOylation during prometaphase compared with other phases, indicating that NKAP SUMOylation predominantly occurred in prometaphase and appeared to be cell-cycle regulated (Fig. 3g).

### SUMOylation is required for NKAP to bind CENP-E

**Figure 4:**
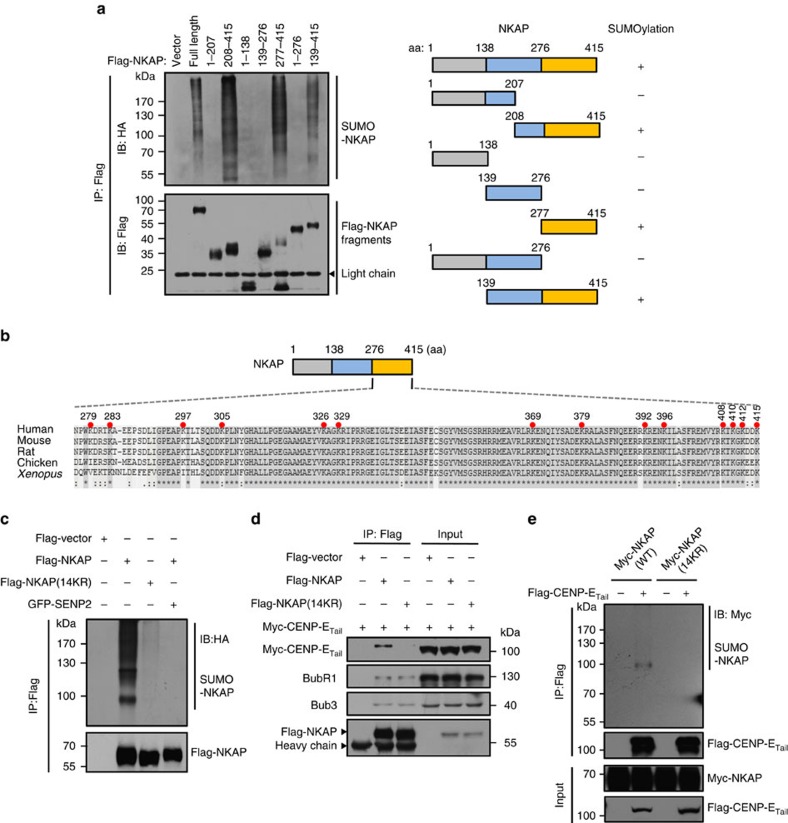
SUMOylation of NKAP is required for its association with CENP-E. (**a**) Left: HEK293T cells were transfected with the indicated Flag-NKAP constructs together with Myc-Ubc9 and HA-SUMO-2 for 48 h. Flag-tagged IPs were immunopurified as described in METHODS and analysed by immunoblot. Right: Schematic representation of full-length NKAP, along with its various deletion mutants. (**b**) Sequence alignments of NKAP C-terminal fragment (aa 276–415) from different organisms using Clustal Omega algorithm. The 14 Lys (K) mutated to Arg (R) in further assays are indicated (red filled circles). All amino acid numbers are for human NKAP. (**c**) *In vivo* SUMOylation assay for NKAP SUMOylation modification sites verification. Flag-NKAP (WT) or Flag-NKAP (14KR) mutant were expressed in HEK293T cells along with HA-SUMO-2 and Myc-Ubc9. The levels of NKAP SUMOylation were evaluated by IP of NKAP using anti-Flag M2 beads followed by anti-HA immunoblot. (**d**) Co-IP analysis of the association of NKAP wild type and SUMO-null mutant with CENP-E. HeLa cells were transfected with the indicated plasmids and arrested in prometaphase. The cell lysates were immunoprecipitated with anti-Flag M2 beads and analysed with the indicated antibodies. (**e**) SUMOylation of NKAP promotes its interaction with CENP-E. HEK293T cells were co-transfected with expression plasmids as indicated. After 48 h of transfection, the cell lysates were subjected to co-IP followed by immunoblot.

Next, we worked on the identification of SUMO modification sites of NKAP. As none SUMO consensus motif was found in NKAP, we generated a series of truncated mutants and tested their SUMOylation. The results indicated that the C-terminal fragment (aa 277–415) was required for NKAP modification by SUMO-2 (Fig. 4a). As depicted in Fig. 4b, there were 14 lysines in this section and notably, they were highly conserved from rat to human, suggesting their important function. When the 14 lysines were mutated to arginines (14KR), the SUMO level of NKAP was almost diminished, indicating that the main SUMO target sites of NKAP were among the 14 lysines (Fig. 4c). We also examined the localization of the NKAP deletion fragments in HeLa cells. The results showed that all the deletion mutants had nuclear localization, no matter whether they could be SUMOylated or not (Supplementary Fig. 5a).

We then examined whether the SUMOylation of NKAP was required for its interaction with CENP-E. We found that the 14KR mutant still interacted with BubR1 and Bub3. In contrast, CENP-E interacted with wild type NKAP much stronger than 14KR mutant (Fig. 4d). A weak interaction of 14KR mutant and CENP-E could be observed, it might be due to the residual SUMOylation of 14KR mutant since a small amount of SUMOylation could still be detected in 14KR mutant (Fig. 4c). Consistently, in the reverse IP experiment using Flag-CENP-E_Tail_ as a bait, CENP-E_Tail_ interacted with the SUMOylated NKAP, but not the 14KR mutant (Fig. 4e). We also used bacterially expressed GST-NKAP to pull-down CENP-E in cell lysates. As shown in Supplementary Fig. 5b, unmodified form of NKAP could pull-down Bub3 but not CENP-E. These results support that the SUMOylation of NKAP promotes its association with CENP-E.

### NKAP SUMOylation is required for CENP-E KT localization

**Figure 5:**
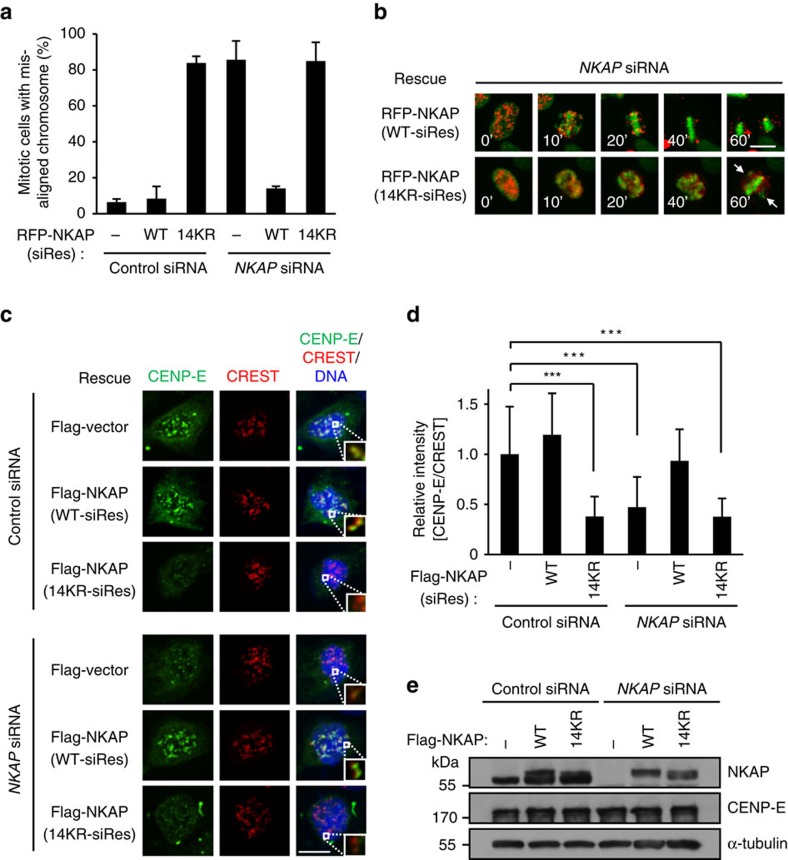
SUMOylation of NKAP is required for the kinetochore localization of CENP-E. (**a**) Expression of siRNA-resistant RFP-NKAP (WT), but not RFP-NKAP (14KR) or RFP vector rescues chromosome misalignment caused by NKAP knockdown. Percentage of mitotic cells with misaligned chromosomes is shown. For quantifications, >100 mitotic cells were counted for each experiment and condition. Data are representative of three independent experiments and shown as mean±s.d. (**b**) Selected frames from time-lapse movies of representative cells as described in **a**. The time on the images is in minutes. Arrowheads indicate unaligned chromosomes. Scale bar, 10 μm. Full frames of all groups are shown in Supplementary Fig. 6a. See Supplementary Movies 4–9. (**c**) Complementation of Flag-NKAP (WT) but not Flag-NKAP (14KR) in NKAP-knockdown cells rescues CENP-E kinetochore localization. HeLa cells were transfected with indicated siRNAs and expressing plasmids for 72 h, followed by nocodazole treatment for 4 h. Cells were stained for CENP-E (green), CREST (red) and DNA (blue). The boxed kinetochore was enlarged to the bottom right of each merged images. Scale bar, 10 μm. (**d**) Quantification of relative fluorescence intensity of the CENP-E signal at kinetochores in cells as described in **c** (*n*>20 cells, >400 kinetochores for each group). Data are shown as mean±s.d. ***P<0.001 (one-way ANOVA). (**e**) Western blot analysis of siRNA-resistant Flag-NKAP (WT), Flag-NKAP (14KR) and endogenous NKAP expression in cells as described in **c**. Cells were randomly selected for observation and statistics.

To further verify the effect of NKAP SUMOylation on chromosome alignment, we performed a rescue experiment with the NKAP 14KR mutant. The time-lapse imaging analysis showed that NKAP-knockdown-induced chromosome misalignment and prometaphase arrest were effectively reversed by wild type NKAP. However, the expression of NKAP 14KR mutant failed to do so (Fig. 5a,b, Supplementary Fig. 6a,b and Supplementary Movies 4–9). Consistently, NKAP-knockdown-induced CENP-E mislocalization was also rescued by wild type NKAP, but not by its 14KR mutant (Fig. 5c–e). In addition, we observed that the 14KR mutant was located on kinetochores, suggesting that NKAP kinetochore localization was independent of its SUMOylation (Supplementary Fig. 6c). Notably, 14KR mutant overexpression resulted in the same misalignment of chromosome and CENP-E mislocalization as NKAP knockdown did, suggesting that the 14KR mutant might function in a dominant negative manner (Fig. 5a,c,d; Supplementary Fig. 6a). Taken together, these results indicate that the SUMOylation of NKAP is required for chromosome alignment and CENP-E localization on kinetochores.

### NKAP localizes to kinetochores through binding Bub3

**Figure 6:**
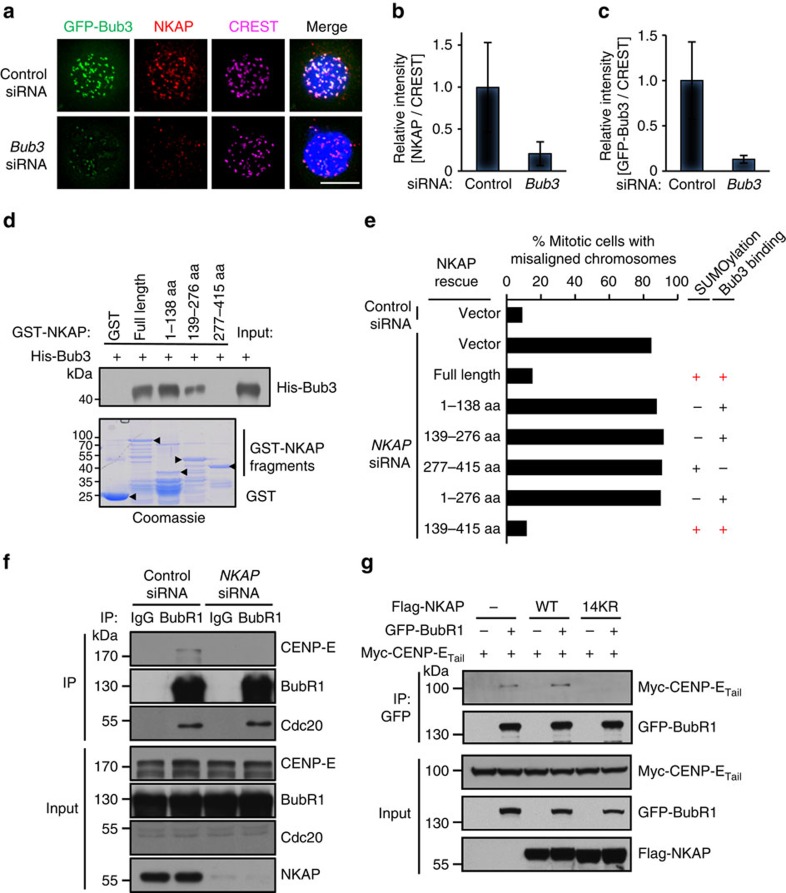
Bub3 recruits NKAP to stabilize BubR1-CENP-E interaction at kinetochores. (**a**) Bub3 knockdown impairs NKAP kinetochore localization. HeLa/GFP-Bub3 cells were treated with control or *Bub3* siRNA for 48 h and synchronized in prometaphase with nocodazole (100 ng ml^−1^) and MG132 (10 μM) for 4 h. The cells were stained with anti-NKAP antibody (red), kinetochores (CREST, magenta) and DNA (blue). Scale bar, 10 μm. (**b**) Quantification of relative fluorescence intensity of the NKAP signal on kinetochores in cells as described in **a**. Fluorescence intensity of NKAP in control cells was normalized to 1 (*n*≥10 cells, >200 kinetochores for each group). Cells were randomly selected for observation and statistics. Data are shown as mean±s.d. (**c**) Knockdown efficiency of siRNA targeting Bub3. (**d**) NKAP directly binds Bub3. Recombinant His-Bub3 protein was incubated with GST, GST-NKAP and its deletion mutants. The bound proteins were determined by immunoblot. The amounts of recombinant proteins from the same reaction were analysed by SDS–PAGE and Coomassie blue staining. Arrowheads indicate the positions of corresponding proteins. (**e**) Deletion analysis of NKAP regions responsible for chromosome alignment. HeLa/GFP-H2B cells were transfected with siRNA-resistant RFP-NKAP constructs for 6 h, preceded by transfection with *NKAP* siRNA for 42 h. The percentage of cells with misaligned chromosomes is quantified for cells expressing each construct. The SUMOylation and Bub3-binding ability of each construct are shown. More than 200 cells were counted in each group. Data are representative of two independent experiments. (**f**) The endogenous association of CENP-E with BubR1 is decreased in NKAP-knockdown cells. HeLa cells were transfected with control or *NKAP* siRNA and synchronized at prometaphase with thymidine–nocodazole treatment. IP was carried out using anti-BubR1 antibody and the IPs were probed with indicated antibodies. (**g**) Wild type NKAP stabilizes the interaction of BubR1 and CENP-E whereas SUMO-null mutant impairs their interaction. GFP-BubR1 and Myc-CENP-E_Tail_ were transfected into HEK293T cells, together with Flag-NKAP or its 14KR mutant. Cells were synchronized in mitosis with thymidine–nocodazole treatment and shaken-off. GFP-BubR1 was immunoprecipitated using anti-GFP resin and probed with indicated antibodies.

In previous experiments, we found that NKAP was associated with kinetochore proteins BubR1 and Bub3 (Fig. 3a; Supplementary Fig. 4a–d). We next investigated whether they contributed to NKAP kinetochore localization. We knocked down BubR1 and Bub3, respectively, and performed immunostaining analyses. The results showed that the NKAP signal intensity on kinetochores was markedly reduced in Bub3-knockdown cells, while it was almost unaffected in BubR1-knockdown cells (Fig. 6a–c; Supplementary Fig. 7a–c). Consistently, compared with the control siRNA-treated cells, the signal intensity of CENP-E on kinetochores was reduced in Bub3-knockdown cells (Supplementary Fig. 7d–f). These results suggest that Bub3 plays a major role in NKAP kinetochore localization. However, since there is still residual of BubR1 in the knockdown cells, the role of BubR1 in NKAP recruitment could not be excluded. The co-IP experiment demonstrated that His-Bub3 was co-IP with Flag-NKAP (Supplementary Fig. 7g). We carried out GST pull-down experiments and found that NKAP directly interacted with Bub3 (Supplementary Fig. 7h). Further mapping analysis revealed that two regions (aa 1–138 and aa 139–276) of NKAP were responsible for its interaction with Bub3 (Fig. 6d). These results suggest that NKAP is recruited to kinetochore through directly binding Bub3.

We next performed rescue experiments with a series of siRNA-resistant deletion mutants of NKAP, and tested whether the interaction of NKAP with Bub3 is essential for its function in chromosome alignment. The time-lapse imaging analysis showed that besides the full-length, the 139–415aa region of NKAP could reverse the chromosome misalignment induced by NKAP depletion (Fig. 6e; Supplementary Fig. 7i). Particularly, the 139–415aa region of NKAP contains both the Bub3-binding region and the SUMOylation region. In contrary, the deletion mutants lacking either the Bub3-binding region or the SUMOylation region could not rescue the chromosome misalignment. These results suggest that both kinetochore localization and SUMOylation are indispensable for NKAP function in chromosome alignment.

### SUMOylated NKAP is essential for CENP-E binding to BubR1

It has been reported by other groups that BubR1 interacts with CENP-E and contributes to CENP-E kinetochore localization^11^,^36^,^37^,^38^,^39^. To investigate whether NKAP plays a role in association of CENP-E with BubR1, we performed co-IP experiments in NKAP-knockdown cells. As shown in Fig. 6f, immunoprecipitating the endogenous BubR1 from nocodazole-arrested mitotic HeLa cells resulted in the co-IP of CENP-E, which is consistent with other groups' results^11^,^12^,^36^,^37^. In contrast, CENP-E could not be detected in the BubR1 immunocomplex when NKAP was knocked down. Meanwhile, the interaction of BubR1 with Cdc20, another known binding protein of BubR1, was not affected by NKAP knockdown. These results suggest that NKAP is required for CENP-E binding to BubR1. We further examined whether NKAP SUMOylation was needed for CENP-E binding to BubR1. As shown in Fig. 6g, the interaction of CENP-E with BubR1 was not observed when the SUMOylation-deficient mutant (14KR) of NKAP was ectopically expressed in HeLa cells. The results were consistent with the dominant negative function of 14KR mutant in CENP-E kinetochore localization and chromosome alignment. Together, these data indicate that SUMOylated NKAP is essential for CENP-E binding to BubR1.

### NKAP loss leads to aneuploidy

**Figure 7:**
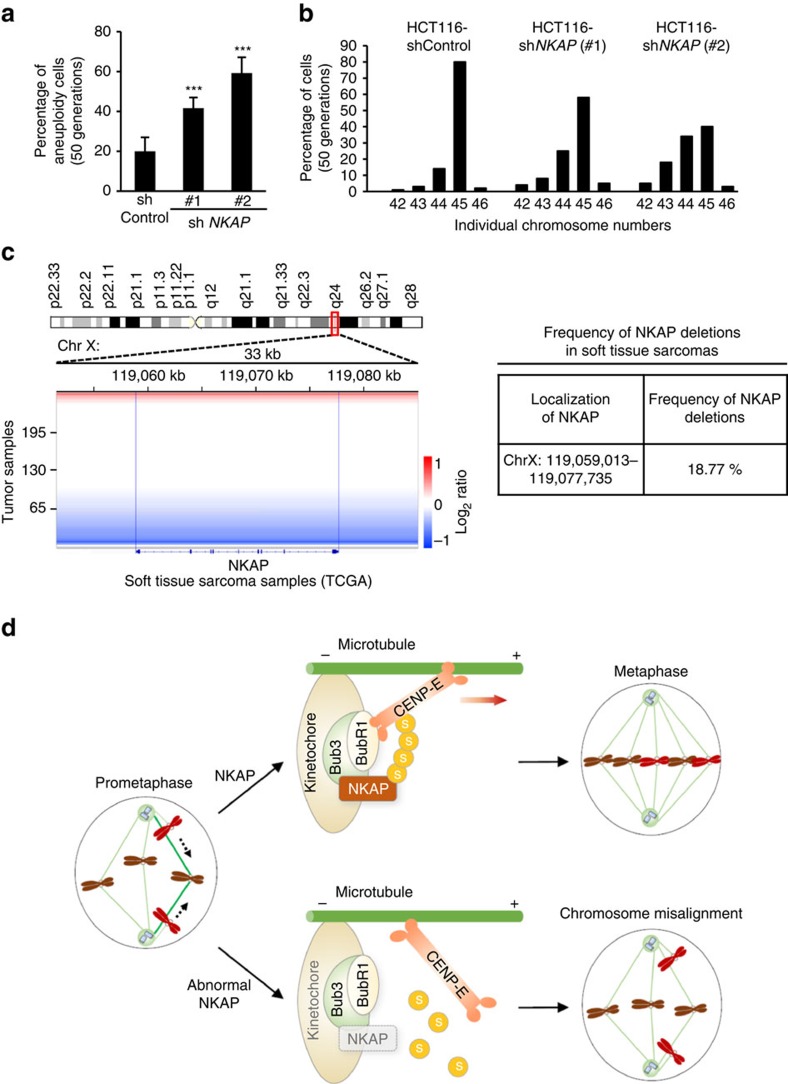
Loss of NKAP leads to aneuploidy and is observed in human sarcomas. (**a**) HCT116 cells were stably infected with lentivirus expressing control or *NKAP* shRNA #1 and #2, and cultured for 50 generations. The proportion of cells with a karyotype deviating from the modal chromosome number was determined within 50 generations in cells as indicated in **b** (*n*>100). Cells were randomly selected for observation and statistics. Data are representative of three independent experiments and shown as mean±s.d. ***P<0.001 (one-way ANOVA). (**b**) Individual chromosome numbers from metaphase spreads of cultured HCT116, HCT116-*NKAP* shRNA #1 and #2 cells were analysed within 50 generations (*n*>100). Cells were randomly selected for observation and statistics. (**c**) Left: SNP array analysis of 261 soft tissue sarcoma samples from TCGA (07/31/2015 data freeze). Segmentation data for the area surrounding *NKAP* on chromosome X are shown. Chromosomal gain or loss is represented by a colour gradient (red, gain; blue, loss). *x* axis represents genomic location along chromosome X. The blue bars represent the boundaries of *NKAP*. Right: The *NKAP* locus is frequently lost in soft tissue sarcomas. Table listing the percentage of soft tissue sarcomas with evidence of *NKAP* deletion (log_2_ ratio <−0.3). (**d**) The model of NKAP regulating CENP-E recruitment to kinetochores during prometaphase. NKAP undergoes SUMOylation in prometaphase and is recruited to kinetochores by Bub3. SUMOylated NKAP interacts with CENP-E and promotes the binding of CENP-E to BubR1, thus stabilizes CENP-E kinetochore localization. When NKAP is deleted or fails to be SUMOylated, CENP-E could not be recruited to kinetochores efficiently. Therefore polar chromosomes fail to be pulled to metaphase plate. As consequence, NKAP plays a critical role in CENP-E kinetochore targeting and chromosome congression.

In the previous time-lapse imaging experiment, higher frequency of chromosome lagging was observed in NKAP-knockdown cells (Fig. 1a). As chromosome lagging could lead to chromosomal instability and aneuploidy^40^,^41^,^42^, we next analyzed the chromosome karyotype in NKAP-depleted HCT116 cells within 30–50 generations. The results showed that NKAP depletion increased the incidences of aneuploidy significantly within 30 generations, and the level of aneuploidy was further elevated after 20 additional generations in NKAP-depleted cells (Fig. 7a,b; Supplementary Fig. 8a–c). These results demonstrated that NKAP depletion-induced chromosomal instability and aneuploidy, suggesting a correlation between NKAP dysfunction and tumorigenesis. Therefore we next asked whether NKAP was dysregulated in human cancers. We analyzed the copy number alterations at the NKAP locus (Xq24) in several human cancers by exploring publicly available datasets of The Cancer Genome Atlas (TCGA). Notably, deletion at the NKAP locus was frequently observed in soft tissue sarcomas, with 18.77% (49/261) of tumours exhibiting loss (log_2_ ratio <−0.3) (Fig. 7c). By exploring the GEO database, the significant decrease of NKAP gene expression was found in other two types of human cancers, pancreatic and thyroid cancer (Supplementary Fig. 8d,e). These data suggest that NKAP is loss of function, in part, via deletion or downregulated in several types of human cancers and support the hypothesis that NKAP deficiency contributes to cancer development by causing genome instability.

## Discussion

Chromosome alignment is a key step for faithful chromosome segregation during mitosis, which is essential for genome integrity^41^,^43^. Understanding how proper chromosome alignment is achieved at the molecular level, is crucial for our understanding of how genome instability develops in tumorigenesis^44^,^45^. Here, we report that NKAP is a key mitotic regulator that plays critical roles in chromosome alignment. NKAP undergoes SUMOylation in mitosis and the SUMOylated NKAP is required for the recruitment of CENP-E to kinetochores (Fig. 7d).

Our works demonstrated that NKAP is associated with several kinetochore proteins, such as Bub3, BubR1 and CENP-E, and plays crucial roles in chromosome alignment. As a large kinetochore-bound motor protein, CENP-E governs dynamic KT–MT interactions and plays critical roles in chromosome alignment. Our work provides a detailed mechanism how NKAP regulates CENP-E recruitment to kinetochores. NKAP is initially recruited to kinetochores in prometaphase. When chromosomes are aligned properly in metaphase, the majority of NKAP disassociate from kinetochores. This dynamic process is similar to that of CENP-E localization on kinetochores. Previous study reported that Bub3 is required for CENP-E kinetochore localization^21^,^38^. We found that NKAP was also recruited by Bub3 to kinetochores. We further demonstrated that NKAP was SUMOylated in mitosis and the SUMOylated NKAP was required for the recruitment CENP-E to kinetochores. It has been reported that BubR1 interacts with CENP-E and contributes to CENP-E kinetochore localization^11^,^36^,^37^,^38^,^39^. Our results indicate that the SUMOylated NKAP is needed to stabilize the binding of CENP-E to BubR1 on kinetochores.

The association of NKAP with CENP-E is regulated by the SUMOylation of NKAP. The structures of SUMO proteins are similar with that of ubiquitin, and they conjugate to substrates through enzymatic cascades^46^. Like ubiquitination, SUMOylation is reversible. The conjugation and de-conjugation of SUMO modification for NKAP could swift rapidly. Since the SUMOylation of NKAP regulates its association with CENP-E, the dynamic SUMOylation of NKAP could be an effective way to fine-tune CENP-E kinetochore localization at the appropriate time. Zhang *et al*.^47^ reported that overexpression of the deSUMOylating enzyme, SENP2, resulted in the dissociation of CENP-E from kinetochores, and demonstrated that CENP-E interacted with SUMO-2/3 polymeric chains by *in vitro* binding assay. Although the substrate is unidentified, they hypothesized that an unknown protein interacts with CENP-E through SUMO-2/3 chains^47^. In this study, consistent with previously high throughput screening studies^48^,^49^,^50^, we identify NKAP as a novel SUMO substrate, and demonstrate that NKAP SUMOylation is needed to form complex with CENP-E. The SUMOylation-deficient mutant of NKAP cannot support the localization of CENP-E on kinetochores or proper chromosome alignment.

The NKAP-regulated CENP-E kinetochore localization is important for chromosomal stability. CENP-E is known to be essential for the maintenance of chromosomal stability. Mice lacking one allele of CENP-E exhibit spontaneous lung adenomas and spleen lymphomas, with their cells high incidences of aneuploidy^51^,^52^. As an upstream regulator of CENP-E, NKAP is required for CENP-E kinetochore localization. Importantly, NKAP depletion consistently results in chromosomal instability and aneuploidy in cultured cells. Particularly, *NKAP* gene deficiency has been observed in soft tissue sarcomas as well as several other types of human cancer. Thus, the NKAP–CENP-E interaction might play essential roles in the maintenance of chromosomal stability.

## Methods

### Plasmids and reagents

*NKAP* inserts were subcloned into pcDNA3.0-Flag, pXJ40-Myc, pIRES2-DsRed, pEGFP-N1 and pGEX-4T-1 vectors. To construct NKAP (14KR) mutants, multi-sites mutated 14KR fragment was synthesized by Integrated DNA Technologies Corporation (Supplementary Methods). To construct RNAi-resistant NKAP, the nucleotide sequence (ACAAGTGAAGAAATTGCA) was changed to (ACcAGcGAgGAgATcGCg). All the mutations were verified by DNA sequencing. Flag-CENP-E_Tail_ was provided by Dr Don Cleveland (University of California, San Diego). HA-SUMO-2, HA-SUMO-1 and GFP-SENP2 were gifts from Dr Xiangdong Zhang (Department of Biological Sciences, Wayne State University). Myc-Ubc9 was gift from Dr Marcos Malumbres (Molecular Oncology Program, CNIO, Madrid, Spain). Lists of all constructs, oligonucleotides and antibodies used in this study are provided in Supplementary Tables 1 and 2. Nocodazole, taxol, N-ethylmaleimide (NEM), phenylmethanesulfonyl fluoride (PMSF) and thymidine were purchased from Sigma. MG132 was from Calbiochem, and ^35^S methionine from Perkin Elmer.

### Cell culture

HeLa cells stably expressing GFP-Bub3 were kindly provided by Xueliang Zhu (Institutes for Biological Sciences, Chinese Academy of Sciences, Shanghai, China), HeLa cells stably expressing GFP-H2B were kindly provided by Stephen Doxsey (University of Massachusetts Medical School, USA) and HeLa cells stably expressing His-SUMO-2 were kindly provided by Ron Hay (Wellcome Trust Centre for Gene Regulation and Expression, University of Dundee, UK). All other mammalian cell lines used in this study were from the American Type Culture Collection. HeLa cells stably expressing RFP-H2B were previously established in our laboratory. All the cell lines have been fully authenticated and tested free of mycoplasma.

### Cell synchronization and transfection

HeLa, HEK293T, U2OS cells were maintained at 37 °C in DMEM (Invitrogen) supplemented with 10% fetal bovine serum and 1% penicillin-streptomycin. HCT116 cells were cultured in RPMI 1640 medium (Invitrogen). For mitotic synchronization, HeLa cells were synchronized by thymidine–nocodazole arrest and shaken-off. G1 phase cells were obtained by releasing mitotic cells into drug-free medium for 4 h. For thymidine–nocodazole arrest, cells were incubated in thymidine-containing (2 mM) medium for 18 h, released into fresh medium for 3 h and treated with nocodazole (100 ng ml^−1^) for 11 h as reported^41^. For detecting NKAP SUMOylation during cell cycle, cells were synchronized by double-thymidine block and collected at the G1/S border (0 h), S phase (4 h) or G2 phase (8 h). Cells were arrested in prometaphase by thymidine–nocodazole treatment and further released into medium containing MG132 (10 μM) for 2 h to get metaphase cells. For plasmid transfection, cells were cultured to 60–70% confluency and transfected with the indicated plasmids using Lipofectamine 2000 Transfection Reagent (Invitrogen). For siRNA transfection, cells were transfected with the indicated siRNA using Lipofectamine RNAiMAX Transfection Reagent (Invitrogen) according to the manufacturer's instructions.

### IP and immunoblot

For BubR1 IP experiment, thymidine–nocodazole-arrested HeLa cells were collected in lysis buffer (50 mM Hepes pH 7.4, 150 mM NaCl, 2 mM EGTA, 0.1% Triton X-100, protease inhibitor cocktail (Roche), 1 mM PMSF). Extracts were cleared by centrifugation (16,000*g*, 10 min at 4 °C) and then incubated with BubR1 polyclonal antibody at 4 °C overnight. After incubation, the beads were washed three times with lysis buffer. Proteins were separated by SDS–PAGE and analyzed by western blot with the indicated antibodies. For other IP experiments, cells were collected in TNE lysis buffer (50 mM Tris–HCl pH 8, 200 mM NaCl, 0.5% NP-40, 0.1 mM EDTA) supplemented with protease inhibitor cocktail (Roche), 1 mM PMSF and 10 mM NEM. IP was carried out with mouse affinity gel against GFP or Flag. All the IP and immunoblot experiments in this study were repeated at least three times. Uncropped scans of typical blots are presented in Supplementary Fig. 9.

### Immunofluorescence

For immunofluorescence analysis, transfected cells were plated on chamber slides or 24-well plates with coverslips (Platinum Line Microscope Cover Glass, Zefon). To visualize kinetochore/centromere protein localization, cells were first treated with siRNA for the indicated time followed by incubation with 10 μM of MG132 for 2 h and then processed for fixation in 4 or 1% paraformaldehyde for 7 min. Next, the cells were incubated with 0.3% Triton X-100 in phosphate-buffered saline (PBS) for 10 min on ice and then blocked in 3% BSA in PBS for at least 1 h. The primary antibody was incubated overnight at 4 °C followed by secondary antibody incubation and DAPI staining. Finally, the cells were mounted by ProLong Gold antifade reagent (Invitrogen).

For NKAP immunostaining assay, cells were first extracted in PHEM buffer (60 mM Pipes, 25 mM Hepes, 10 mM EGTA, 2 mM MgCl_2_, pH 6.9) plus 1% Triton X-100 for 5 min. Then cells were fixed with 4% formaldehyde in PHEM buffer for 20 min at room temperature. The extracted cells were blocked in 3% BSA in TBS-TX (20 mM Tris pH 7.5, 150 mM NaCl and 0.1% Triton X-100) for at least 1 h followed by incubation with primary and secondary antibodies. The cells were mounted by ProLong Gold antifade reagent (Invitrogen) and the images were collected by using DeltaVision Deconvolution Microscope (GE Healthcare) with 60 × oil immersion objective lens. SoftWoRx software was used for image deconvolution and analysis. The optical sections were taken at intervals of 0.2 μm and images were presented as maximal intensity projections. Image analysis was performed using ImageJ software (National Institutes of Health). All the immunofluorescence experiments in this study were repeated at least three times.

### Quantification of immunofluorescence data

To quantify the fluorescence intensity of kinetochore/centromere protein, images were taken from the stacks of images using UltraView spinning-disc confocal microscope (Perkin Elmer) or DeltaVision Deconvolution Microscope (GE Healthcare). The optical sections were taken at intervals of 0.2 μm, and slices of kinetochore images were merged by using the Maximum Intensity Mode. Kinetochore signal intensity was determined by measuring integrated fluorescence intensity with a 10 × 10 pixel square. Background signal was subtracted from an area adjacent to the kinetochore. The images were collected by using identical imaging settings, and the signal of CREST staining was used as reference. The protein fluorescence intensity was normalized to the CREST level of the corresponding kinetochore pair. The relative fluorescence intensity was obtained by normalizing the average intensity from aligned kinetochores to 1. Kinetochore fluorescence intensity analysis was performed using ImageJ software (National Institutes of Health)

To determine the inter-kinetochore distance, cells were fixed with 4% paraformaldehyde, permeabilized with 0.3% Triton X-100 in PBS, and processed with primary and secondary antibodies as described above. Monoclonal antibodies against α-tubulin (Sigma) were used to stain for mitotic spindle. Anti-human centromere antibodies (CREST, Antibodies) were used at 1:100 dilution to visualize centromeres. The centre-to-centre distances between sister kinetochores were measured from different confocal image stacks. When sister kinetochores were in the same focal plane, the inter-kinetochore distance were measured. Images were collected by using an UltraView spinning-disc confocal microscope and analyzed by ImageJ software (National Institutes of Health). To determine the stability of kinetochore fibres, we treated HeLa cells with MG132 (10 μM) for 2 h, washed and then incubated cells with pre-cold Opti-MEM (Thermo Fisher) media on ice for 5 min. Then cells were fixed and permeabilized as described above. The percentage of kinetochores without microtubule attachment was analyzed by IMARIS (Bitplane). The mean background of α-tubulin-stained microtubule (green) was determined by manually placing 20–30 spheres with a diameter of 0.7 μm outside the mitotic spindle. The intensities of CREST-stained kinetochores (red) were automatically detected using the IMARIS spot detection. The diameter of kinetochore sphere to be detected was set as 0.7 μm, and the intensity of microtubules within this sphere was determined. The mean background was subtracted, and the values below background were considered as kinetochores without attachment.

### SUMOylation assay *in vivo*

For *in vivo* SUMOylation assay, cells were sonicated in RIPA buffer (50 mM Tris–HCl, pH 8.0, 150 mM NaCl, 1% NP-40, 0.5% sodium deoxycholate, 0.1% SDS, supplemented with 20 mM NEM, protease inhibitor cocktail (Roche)) and the lysates were heated at 95 °C for 10 min. Following cooling on ice, cell lysates were diluted with the same buffer without SDS to adjust the SDS concentration to 0.1%. Flag-tagged IPs from cell lysis with anti-Flag M2 beads were analyzed by immunoblot with indicated antibodies.

HeLa cells stably expressing His-SUMO-2 were transfected with or without Myc-Ubc9 and Flag-NKAP. A parental HeLa cell was used as a negative control. Forty-eight hours after transfection, cells were lysed in 1 ml of lysis buffer (6 M guanidinium HCl, 100 mM Na_2_HPO_4_, 100 mM NaH_2_PO_4_ and 10 mM Tris–HCl (pH 7.8)). After sonication, 90% of the lysates were incubated with 50 μl Ni-NTA agarose beads (Qiagen). The beads were washed twice with washing buffer (10 mM Tris–HCl (pH 7.8), 100 mM sodium phosphate buffer pH 8.0, 0.1% Triton X-100, 5 mM β-mercaptoethanol) containing 8 M urea, followed by three times washing with washing buffer (pH 6.3) containing 8 M urea. After a final washing with PBS, the beads were treated in SDS sample buffer for SDS–PAGE. The proteins were analyzed by western blot using the indicated antibodies. All the SUMOylation related experiments were repeated at least three times.

### *In vitro* binding assay

All GST-fusion proteins were expressed in *Escherichia coli* DH5α cells. GST, GST-NKAP (wild type) and different NKAP truncation proteins were purified by glutathione Sepharose 4B chromatography according to the manufacturer's protocol (Amersham Biosciences). ^35^S-labelled Flag-Bub3 was translated *in vitro* with a TnT Quick Coupled Transcription/Translation Systems (Promega). The human His-Bub3 full-length recombinant protein was purchased from Abcam (ab139802). The *in vitro* translated Bub3 or recombinant His-Bub3 was incubated with GST or GST-fusion proteins in the binding buffer (50 mM Tris–HCl, pH 7.5, 150 mM NaCl, 1 mM EDTA, 0.3 mM dithiothreitol, 0.1% NP-40, and protease inhibitor cocktail (Roche)). The binding reaction was rocked overnight at 4 °C, and the beads were subsequently washed four times with the binding buffer. The bound proteins were then boiled and separated by SDS–PAGE. All the GST pull-down experiments were repeated at least three times.

### Time-lapse imaging

HeLa/GFP-H2B and HeLa/RFP-H2B stable cell lines were seeded on an eight-chambered cover glass (Lab-Tek Chambered no 1.0 Borosilicate Cover Glass System, Nunc) in CO_2_-independent DMEM (Gibco). Images were collected every 5 min using a 0.1 s exposure for 20 h using a 40 × (or 20 × ) lens objective on inverted fluorescence microscope (Nikon Eclipse Ti-E) with an UltraView spinning-disc confocal scanner unit (Perkin Elmer). The temperature of the imaging medium was kept at 37 °C. Image sequences were viewed using Volocity software, and the cell behaviours were analysed manually. All the time-lapse experiments were repeated at least three times.

### Gel filtration

Mitotic HeLa cells were lysed in NP-40-lysis buffer (PBS with 0.5% NP-40, 10 mM NEM, and protease and phosphatase inhibitors) and subjected to gel filtration. Before gel filtration, lysates were cleared by centrifugation (4 °C) at 16 ,000 g for 30 min. Protein extract (5 mg) was then loaded on a Superose 6 10/300 GL column (GE Healthcare). The isocratic elution was performed at a flow rate of 0.5 ml min^−1^ at 4 °C with PBS, and the fractions were collected every 0.5 ml. Proteins in each column fraction were concentrated by TCA (13%) precipitation and analyzed by immunoblot. The gel filtration experiments were repeated twice.

### Protein identification by mass spectrometry analysis

The protein bands were excised from the SDS–PAGE gel and digested in gel with trypsin. The digested peptides were desalted and analyzed using a Triple TOF 5600^+^ mass spectrometer (AB Sciex, Concord, Canada) coupled to an Ekspert NanoLC 425 (Eksigent, Dublin, OH, USA). Auto calibration was performed every two samples (2 h) to assure high-mass accuracy in both MS and MS/MS acquisition. All Mascot searches were performed against a downloaded SwissProt database (released in Jan 2015, 547, 357 proteins, 194, 874, 700 residues) with taxonomy of homo sapiens, using the following settings: trypsin with up to two missed cleavages; carbamidomethylation fixed at Cys; variable oxidation at Met; variable protein N-terminal acetylation, variable deamidation at Asn and Gln; The mass error tolerance for precursor and fragment ions was set to 20 p.p.m. and 0.2 Da. A decoy reverse database search was also performed to estimate the false-positive rate of protein identification. For unambiguous identification of proteins, the false discovery rate was set to 1%, and a matching of at least two peptides, each with more than 28 ion score. The mass spectrometry analyses were repeated twice.

### Lentivirus infection

The lentivirus constructs stably expressing shRNA-NKAP or shRNA-control was introduced into HCT116 cells using lentivirus-mediated transduction. The cells were harvested, washed and plated in each well of a 6-well plate (400 cells) in complete media. After 10 days, the colonies were collected and detected for knockdown effect.

### RNA interference

*NKAP* siRNA #1 was from Invitrogen (HSS128451) and *NKAP* siRNA #2 was from Invitrogen (HSS188063): target sequences, 5′-GAAGAGTCCGAAGCCCAGCAAATCT-3′ and 5′-GACAAGTGAAGAAATTGCATCATTT-3′, respectively. Control siRNA against *Photinus pyralis luciferase* gene (Invitrogen), target sequence 5′-GGAUUUCGAGUCGUCUUAAUGUAUA-3′; *CENP-E* siRNA (Invitrogen, HSS173791), target sequence 5′- CGGCTCAAGGAAGGCTGTAATATAA-3′; *BubR1* siRNA (Invitrogen, HSS101140), target sequence 5′-AAAUGGAGAAAGGUACACUGGGACC-3′; *Bub3* siRNA (RIBOBIO), target sequence 5′- AACAAAAAGCGACTGTGCCAA-3′. The lentivirus construct for *NKAP* shRNA #1 and #2 were purchased from Sigma MISSION shRNA (TRCN0000145605) and (TRCN0000121619), target sequences are 5′-CTGATTGTCCAGAAGACATTT-3′ and 5′-GAAGAAGTCTAGCCGTTCAAA-3′, respectively.

### qRT-PCR

Total RNAs were extracted with Trizol reagent (Invitrogen) and subjected to oligo (dT)-primed reverse transcription using the PrimeScript RT reagent Kit. The quantitative PCR was performed using an Applied Biosystems 7,300 Real-Time PCR System. All reactions were performed in triplicate. GAPDH served as an internal control.

### Chromosome aneuploidy and spread analyses

After treated with 50 ng ml^−1^ colchicine for 6 h, cells were collected and hypotonically swollen in 75 mM KCl for 45 min at 37 °C. Cells were then fixed in freshly made Carnoys fixative solution (75% methanol–25% acetic acid). Cells were dropped onto cooled glass slides and dried at room temperature. Chromosomes were stained in 5% Giemsa for 10 min, rinsed with PBS, air dried and mounted. The chromosome aneuploidy and spread analyses were repeated three times.

### CNV analysis

The level 3 CNV data from the BI Genome-Wide SNP 6 platform of Sarcoma were downloaded from TCGA (updated 2015/07/31). A total of 261 tumour segmentation files were used for analysis, including 257 primary solid tumours, 3 recurrent solid tumours and 1 metastatic cancer. We used the log ratio 2 (Segment_Mean) to determine copy number change and used thresholds ≥0.3 for gain and ≤−0.3 for loss. We used IGV to show the segmentation data surrounding NKAP on chromosome Xq24 (ref. 53).

### Statistics

Statistical comparisons between only two groups were carried out by Student's *t*-test or the Mann–Whitney *U* test when a normal distribution could not be assumed. One-way ANOVA was used for multiple-group comparisons. SPSS 13.0 was used for the analysis of statistical significance.

### Data availability

The authors declare that the data supporting the findings of this study are available on request from the corresponding authors (Q.X. or X.Z.).

## Additional information

**How to cite this article:** Li, T. *et al*. SUMOylated NKAP is essential for chromosome alignment by anchoring CENP-E to kinetochores. *Nat. Commun.* **7,** 12969 doi: 10.1038/ncomms12969 (2016).

## Supplementary Material

Supplementary Information Supplementary Figures 1-9, Supplementary Tables 1-2, Supplementary Methods and Supplementary References.

Supplementary Data 1 Result of siRNA screening for mitotic regulators

Supplementary Movie 1 Control cells showed normal mitotic progression. HeLa/GFP-H2B cells were treated with control siRNA. After 48 hr, cells were incubated in medium containing 2 mM thymidine for 18 hr, and then released into fresh medium for 7 hr. Images were acquired at 5 min intervals.

Supplementary Movie 2 NKAP siRNA #1-treated cells showed marked delay of anaphase onset. HeLa/GFP-H2B cells were treated with NKAP siRNA #1. After 48 hr, cells were incubated in medium containing 2 mM thymidine for 18 hr, and then released into fresh medium for 7 hr. Images were acquired at 5 min intervals.

Supplementary Movie 3 NKAP siRNA #2-treated cells showed marked delay of anaphase onset. HeLa/GFP-H2B cells were treated with NKAP siRNA #2. After 48 hr, cells were incubated in medium containing 2 mM thymidine for 18 hr, and then released into fresh medium for 7 hr. Images were acquired at 5 min intervals.

Supplementary Movie 4 Control cells showed normal mitotic progression. HeLa/GFP-H2B cells were transfected with RFP empty vector and control siRNA. After 48 hr, cells were incubated in medium containing 2 mM thymidine for 18 hr, and then released into fresh medium for 7 hr. Images were acquired at 10 min intervals.

Supplementary Movie 5 NKAP overexpression cells showed normal mitotic progression. HeLa/GFP-H2B cells were transfected with NKAP siRNA resistant RFP-NKAP (WT) and control siRNA. After 48 hr, cells were incubated in medium containing 2 mM thymidine for 18 hr, and then released into fresh medium for 7 hr. Images were acquired at 10 min intervals.

Supplementary Movie 6 RFP-NKAP (14KR) overexpression caused prometaphase arrest in cells. HeLa/GFP-H2B cells were transfected with NKAP siRNA resistant RFP-NKAP (14KR) and control siRNA. After 48 hr, cells were incubated in medium containing 2 mM thymidine for 18 hr, and then released into fresh medium for 7 hr. Images were acquired at 10 min intervals.

Supplementary Movie 7 NKAP knockdown caused prometaphase arrest in cells. HeLa/GFP-H2B cells were transfected with RFP empty vector and NKAP siRNA. After 48 hr, cells were incubated in medium containing 2 mM thymidine for 18 hr, and then released into fresh medium for 7 hr. Images were acquired at 10 min intervals.

Supplementary Movie 8 RFP-NKAP (WT) rescued prometaphase arrest in NKAP-knockdown cells. HeLa/GFP-H2B cells were transfected with NKAP siRNA resistant RFP-NKAP (WT) and NKAP siRNA. After 48 hr, cells were incubated in medium containing 2 mM thymidine for 18 hr, and then released into fresh medium for 7 hr. Images were acquired at 10 min intervals.

Supplementary Movie 9 RFP-NKAP (14KR) failed to rescue prometaphase arrest in NKAP-knockdown cells. HeLa/GFP-H2B cells were transfected with NKAP siRNA resistant RFP-NKAP (14KR) and NKAP siRNA. After 48 hr, cells were incubated in medium containing 2 mM thymidine for 18 hr, and then released into fresh medium for 7 hr. Images were acquired at 10 min intervals.
