# Supplementary material for: SUMOylated NKAP is essential for chromosome alignment by anchoring CENP-E to kinetochores
Source: Nat Commun. 2016 Oct 3;7:12969. doi: 10.1038/ncomms12969 (PMC5064014; doi:10.1038/ncomms12969)
Supplement: Supplementary Information — Supplementary Figures 1-9, Supplementary Tables 1-2, Supplementary Methods and Supplementary References. [file ncomms12969-s1.pdf]

Supplementary Figures and Legends

Supplementary Figure 1

a

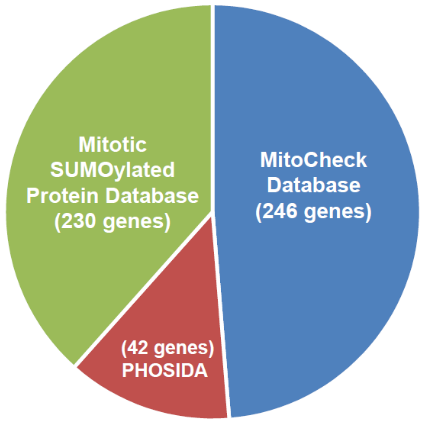

b

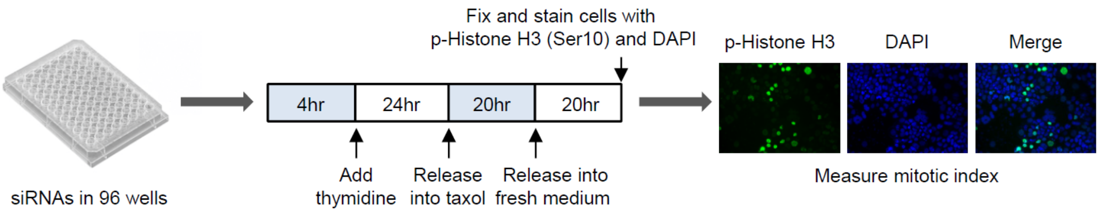

c

Gene Name	Gene ID	Mitotic Index(%)
AUP1	550	37.305
CUEDC1	404093	36.093
NKAP	79576	35.331
LOC137886	137886	30.279
FLJ25555	124930	29.655
UBE2F	140739	29.278
GGA2	23062	28.664
DHX57	90957	27.486
N4BP2	55728	26.492
KIAA0999	23387	25.773
GGA3	23163	25.345
NSFL1C	55968	22.611

Supplementary Figure 1 Identification of NKAP as a novel mitotic regulator through siRNA screening. (**a**) Composition of the 518 mitotic regulator candidate genes. Blue part: 246 genes from Mitocheck database (http://www.mitocheck.org)[1];

Green part: 230 genes from cell cycle-related SUMOylated protein database established by M. W. Kirschner's group[2]; Red part: 42 genes from phosphorylation site database (PHOSIDA) established by Matthias Mann's group[3]. The candidate genes are implied in these databases, but have never been proven to be involved in cell cycle regulation previously. (**b**) Schematic of siRNA-based screen for mitotic regulator. siRNAs targeting candidate genes were transduced into HeLa/GFP-H2B cells. Cells were synchronized as depicted and assayed for mitotic index. p-Histone H3 was used as mitotic indicator. (**c**) List of partial candidate genes with high mitotic index.

Supplementary Figure 2

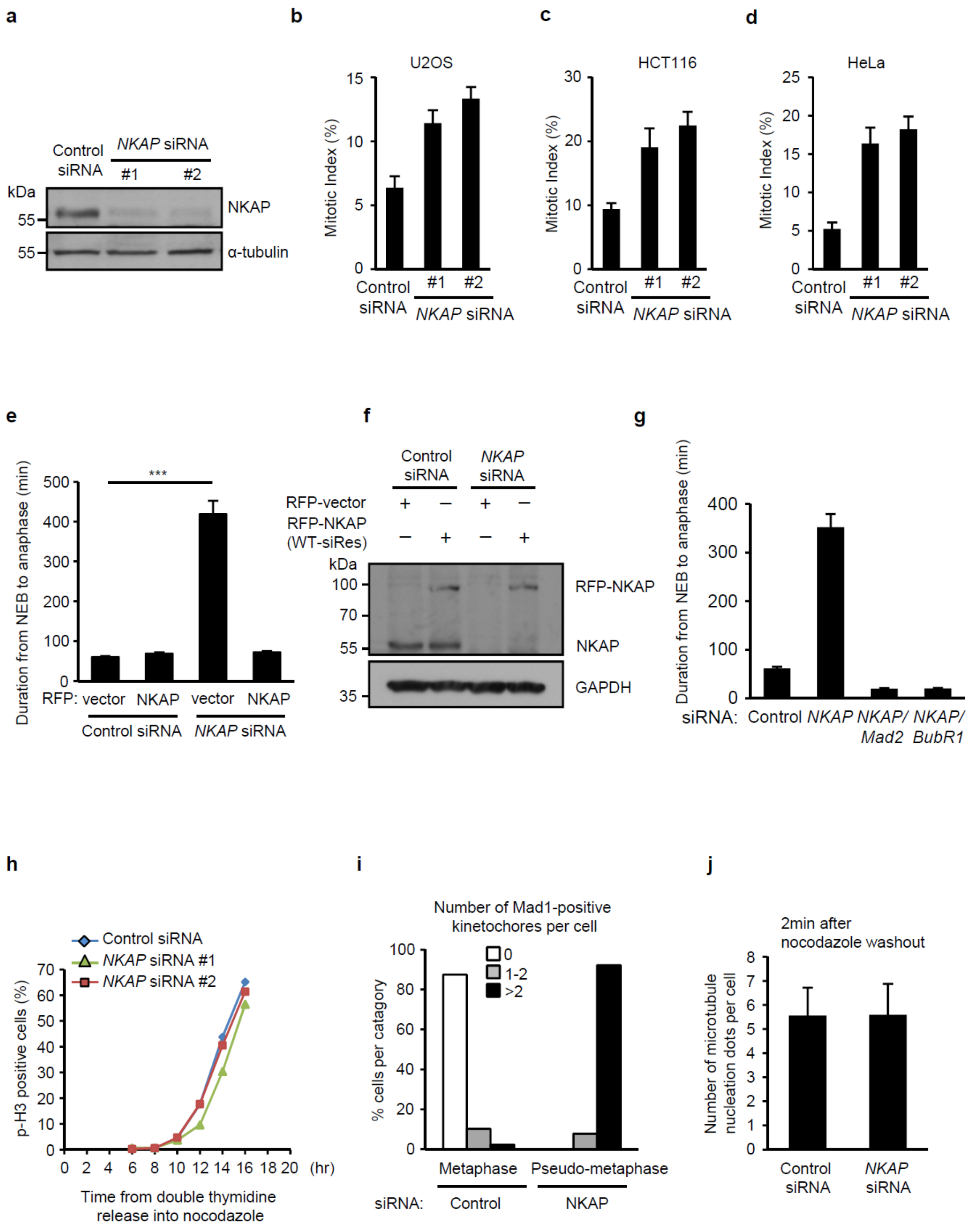

Supplementary Figure 2 NKAP depletion causes prometaphase arrest. (**a**) Immunoblot analysis of cells transfected with control siRNA and *NKAP* siRNAs as described in Fig. 1a. (**b-d**) NKAP knockdown increases mitotic index in U2OS (**b**), HCT116 (**c**) and HeLa cells (**d**). Data are representative of three independent experiments and shown as mean ± s.d. (n > 600 cells). (**e**) Complementation of RFP-NKAP in NKAP-knockdown HeLa/GFP-H2B cells rescues anaphase onset. The

quantification was made from the same experiment as described in Fig. 1e. Data are representative of three independent experiments and shown as mean ± s.e.m. *** $P <$ 0.001 (One-way ANOVA). (**f**) Western blot analysis for the expression of *NKAP* siRNA-resistant RFP-NKAP and endogenous NKAP in cells as described in Fig. 1e. (**g**) Duration time from NEB to anaphase onset in HeLa/GFP-H2B cells treated with the indicated siRNAs. More than 50 cells were counted for each group. Data are mean ± s.e.m. (**h**) NKAP knockdown has little effect on the mitotic entry. HeLa cells transfected with control or *NKAP* siRNAs were synchronized in G1/S by thymidine treatment and then released into nocodazole. Cells were collected at the indicated times after nocodazole treatment and analysed by FACS. p-Histone H3 (Ser-10) acts as an indicator of M phase. (**i**) Quantification of Mad1-positive kinetochore number per cell was made from the same experiment as described in Fig. 1h. n>80 cells for control and *NKAP* siRNA group. (**j**) Microtubule regrowth assay for control and NKAP-depleted cells. HeLa cells were treated with the indicated siRNAs before nocodazole arrest. The number of microtubule nucleation dots in mitotic cells was counted at 2 min after nocodazole washout. Data are representative of three independent experiments and shown as mean ± s.d. Cells were randomly selected for observation and statistics.

Supplementary Figure 3

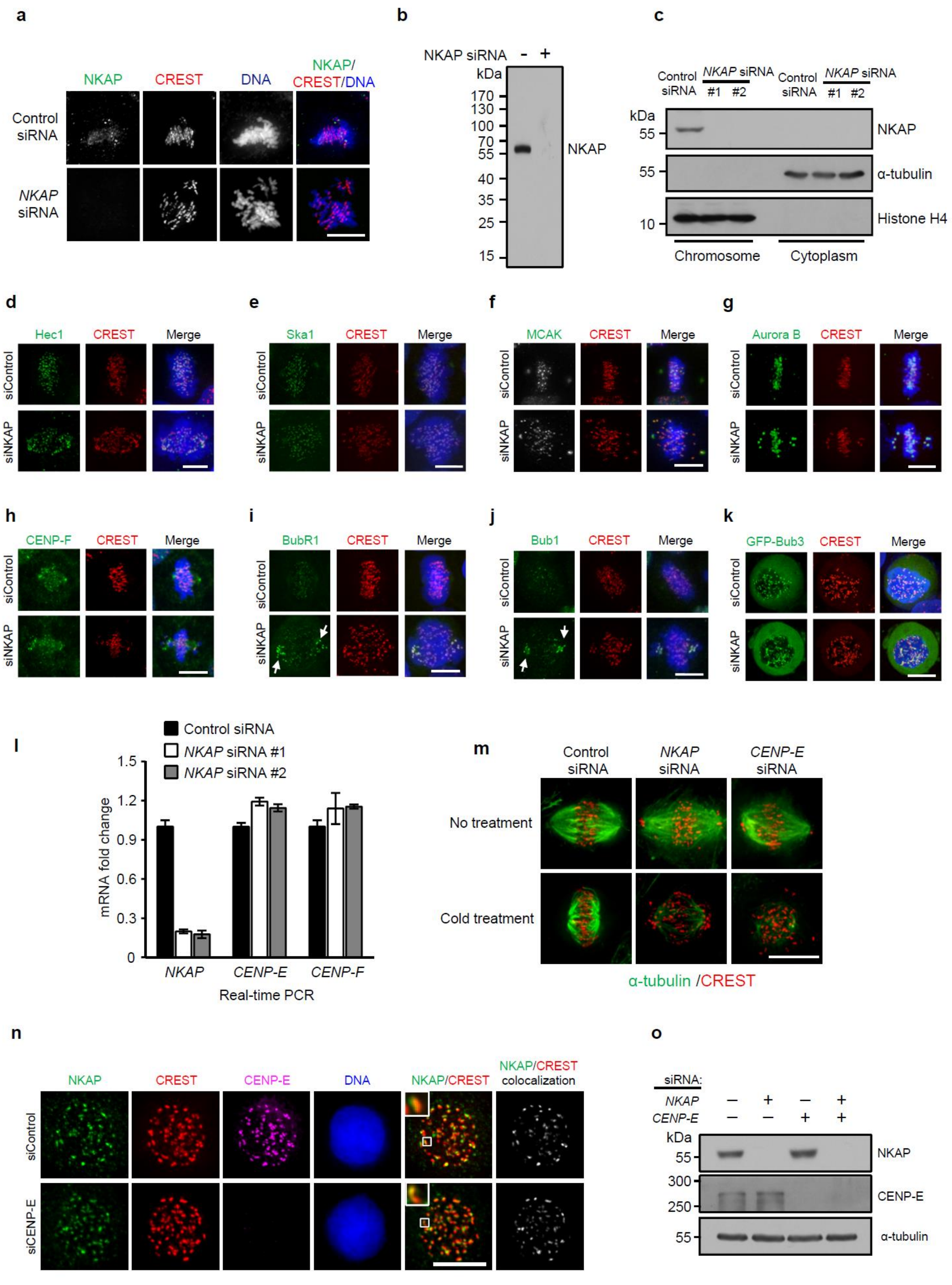

Supplementary Figure 3 NKAP depletion impairs CENP-E kinetochore localization. (**a**) Validation of the NKAP antibody (mAb A2-5) for immunofluorescence staining. HeLa cells were transfected with control or *NKAP* siRNA, and then subjected

to immunofluorescence staining with NKAP antibody (green), CREST antisera (kinetochores, red) and DAPI (DNA, blue). Scale bar, 10 μm. (**b**) Validation of the specificity of anti-NKAP antibody (mAb A2-5) by western blot. (**c**) Chromosome isolation of mitotic cells transfected with control or *NKAP* siRNA. The chromosome fraction and supernatant were subjected to immunoblot analysis with the indicated antibodies. (**d-j**) Immunofluorescence staining for the indicated proteins in control or NKAP-depleted cells. HeLa cells were transfected with control or *NKAP* siRNA for 48 hr and then treated with MG132 (10 μM) for 2 hr. Cells were stained with antibodies for the indicated proteins (green), together with kinetochores (CREST, red) and DNA (blue). Scale bars, 10 μm. (**k**) NKAP knockdown has little effect on Bub3 kinetochore localization. HeLa/GFP-Bub3 cells were treated as described in d. Scale bar, 10 μm. (**l**) Real-time PCR analysis of NKAP, CENP-E and CENP-F mRNA levels in control and NKAP-knockdown cells. (**m**) CENP-E knockdown leads to KT-MT attachment instability similarly as NKAP knockdown does. HeLa cells were transfected with the indicated siRNAs and treated as described in Fig. 1f. Scale bar, 10 μm. (**n**) CENP-E knockdown does not affect NKAP kinetochore localization. The control and CENP-E-depleted cells were treated with nocodazole for 4 hr and stained with antibodies for NKAP (green), kinetochores (CREST, red), CENP-E (magenta) and DNA (blue). The boxed regions are magnified to show typical NKAP localization on kinetochores. Scale bar, 10 μm. (o) Western blot analysis of the indicated proteins in cells as described in Fig. 2h.

Supplementary Figure 4

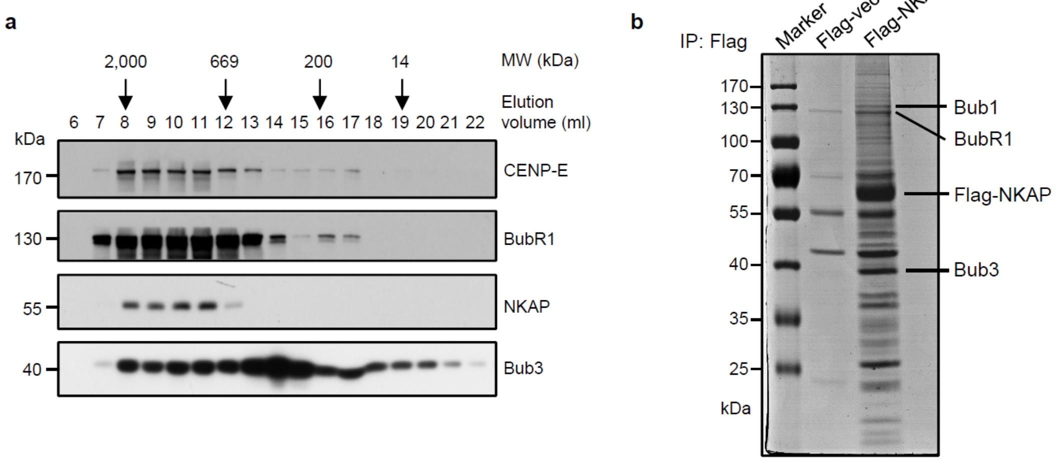

c

NKAP interacting proteins on kinetochores identified by MS/MS analysis

**Protein name**	**MW (kDa)**		**score**
	Theory	observed	
Bub3	37.59	40.21	1183
Bub1	123.44	130.12	244
BubR1	120.78	127.03	57

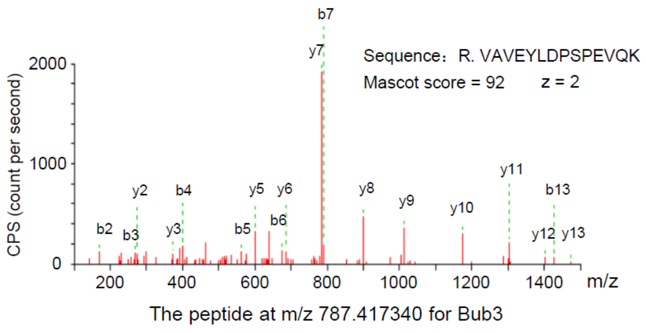

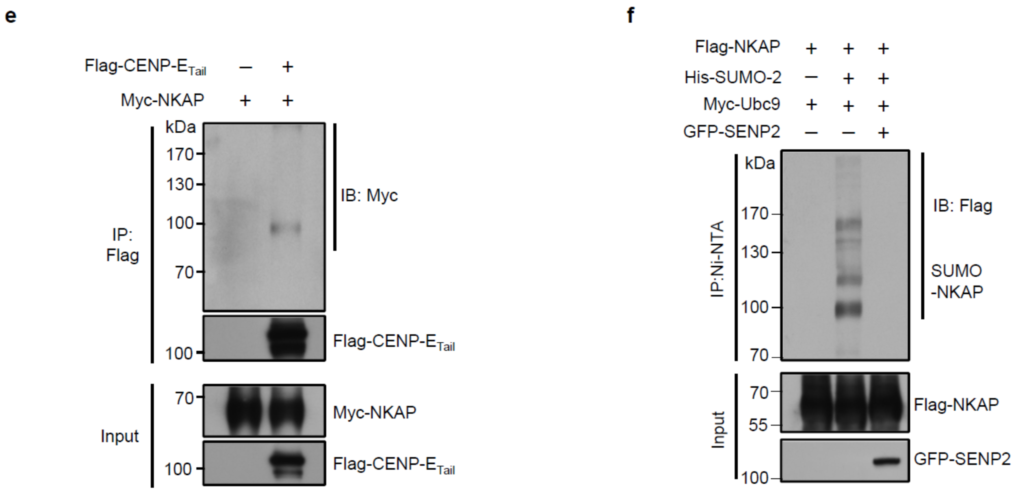

Supplementary Figure 4 NKAP is associated with kinetochore proteins. (**a**) Lysates from thymidine-nocodazole treated HeLa cells (shake-off) were fractionated

on a Superose 6 gel filtration column and blotted with the indicated antibodies. The elution volume and the native molecular mass standards are indicated. (**b**) Flag-NKAP IPs were separated by SDS-PAGE and stained with Coomassie blue. The Flag-NKAP interacted protein bands were cut out and subjected to trypsin digestion and mass spectrometry analysis. The bands for the identified proteins listed in **c** were indicated. (**c**) Kinetochore proteins Bub1, Bub3 and BubR1 were identified as NKAP-interacting proteins by mass spectrometry analysis. (**d**) Identification of Bub3 by MS after trypsin digestion. The peptide at m/z 787.417340 for Bub3 was sequenced by NanoLC-MS/MS, and the deduced sequence was indicated. (**e**) HEK293T cells were co-transfected with vectors expressing Flag-CENP-E_{Tail} and Myc-NKAP. The cell lysates were immunoprecipitated with anti-Flag M2 beads and blotted with anti-Myc antibody. (**f**) SUMOylation assay for NKAP in HeLa/His-SUMO-2 and parental cells. Cells were transfected with plasmids as indicated and arrested in prometaphase with nocodazole treatment. His-SUMO-2 conjugates were recovered on Ni-NTA beads and analysed by immunoblot with the indicated antibodies.

Supplementary Figure 5

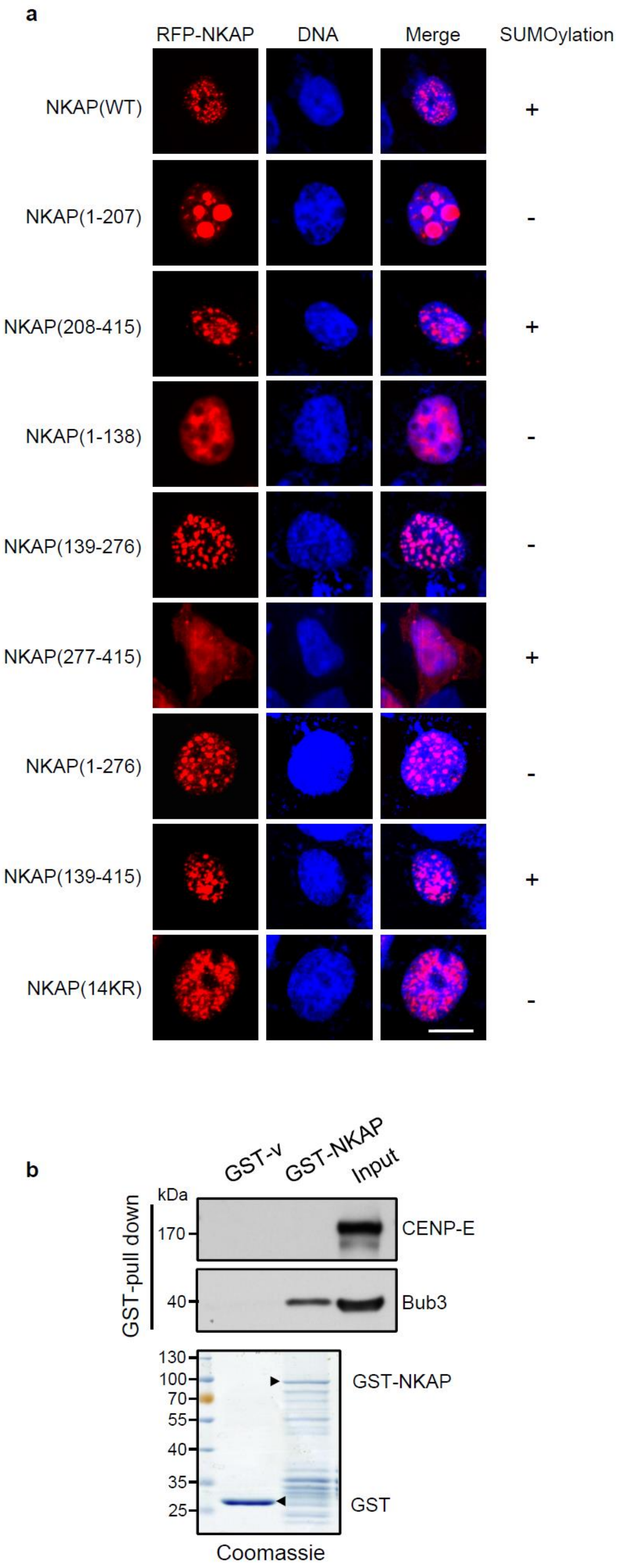

Supplementary Figure 5 Non-SUMOylated NKAP fails to interact with CENP-E. (**a**) Localization of the RFP-NKAP WT, 14KR mutant and deletion fragments in HeLa cells. Scale bars, 10 µm. (**b**) Unmodified form of NKAP could pull down Bub3 but not CENP-E. Bacterially expressed GST-NKAP was used to pull down its interacting proteins in mitotic lysates from HeLa cells. The bound proteins were determined by immunoblotting with the indicated antibodies.

Supplementary Figure 6

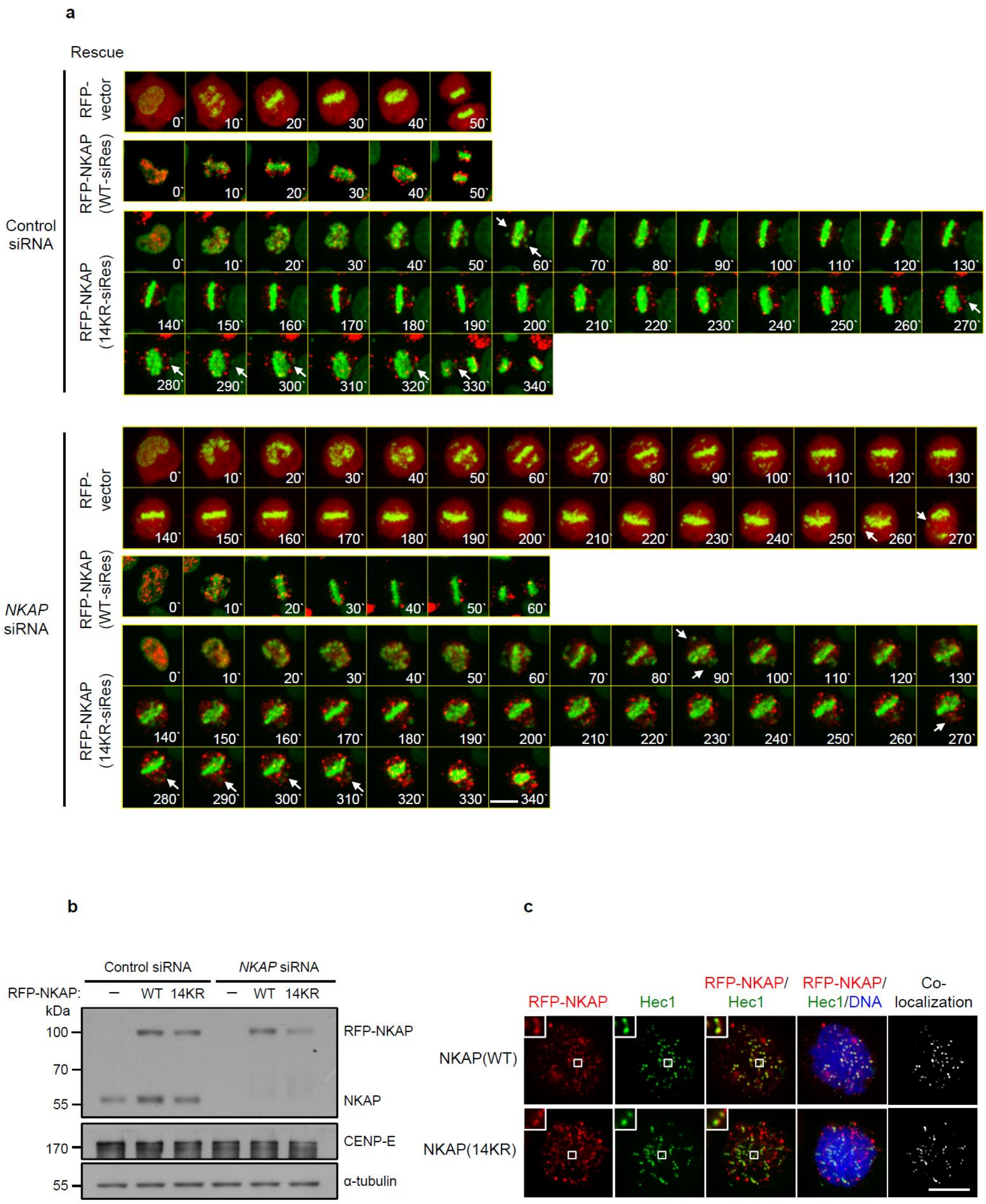

Supplementary Figure 6 SUMOylation of NKAP is required for chromosome alignment. (**a**) Complementation of RFP-NKAP in NKAP-knockdown HeLa/GFP-H2B cells rescues prometaphase arrest and chromosome misalignment. HeLa/GFP-H2B cells depleted of endogenous NKAP were transfected with RFP empty vector, *NKAP* siRNA resistant RFP-NKAP (WT) or RFP-NKAP (14KR) plasmids. Selected frames

from time-lapse movies of representative cells are shown. The time on the images is in minutes. Arrows indicate the misaligned or lagging chromosomes. Scale bar, 10 µm. See Supplementary Movies 4-9. (**b**) Expressions of *NKAP* siRNA-resistant RFP-NKAP (WT), RFP-NKAP (14KR) and endogenous NKAP were examined by western blot. (**c**) Both RFP-NKAP (WT) and RFP-NKAP (14KR) show kinetochore localization in prometaphase. Cells were stained for Hec1 (green) and DNA (blue). The boxed kinetochores was enlarged to the upper left of the images. Scale bar, 10 µm.

Supplementary Figure 7

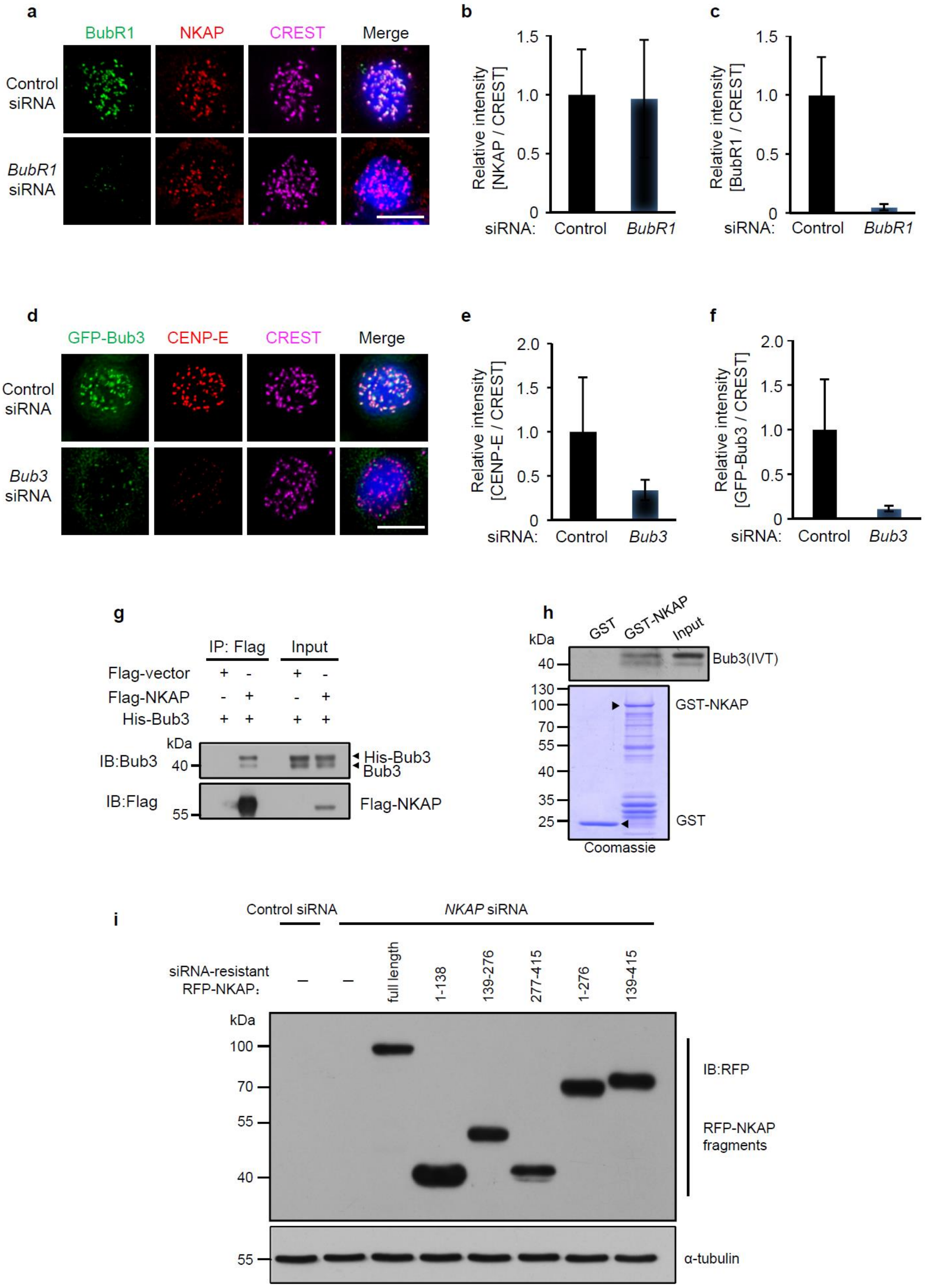

Supplementary Figure 7 NKAP is associated with Bub3. (**a**) BubR1 knockdown has little effect on NKAP kinetochore localization. HeLa cells were treated with control or *BubR1* siRNA for 48 hr and synchronized in prometaphase with nocodazole (100

ng/ml) and MG132 (10 µM) for 4 hr. The cells were stained for NKAP (red), BubR1 (green), CREST (magenta) and DNA (blue). Scale bar, 10 µm. (**b**) Quantification of relative fluorescence intensity of the NKAP signal on kinetochores in cells as described in **a**. Fluorescence intensity of NKAP in control cells was normalized to 1 (n ≥ 10 cells, >150 kinetochores for each group of each experiment). Data are shown as mean ± s.d. (**c**) Knockdown efficiency of siRNA targeting BubR1. (**d**) Bub3 knockdown reduces CENP-E kinetochore localization. Cells were treated as described in Fig. 6a. The cells were stained for CENP-E (red), CREST (magenta) and DNA (blue). Scale bar, 10 µm. (**e**) Quantification of relative fluorescence intensity of the CENP-E signal on kinetochores in cells as described in **d**. Fluorescence intensity of CENP-E in control cells was normalized to 1 (n ≥ 10 cells, >150 kinetochores for each group of each experiment). Cells were randomly selected for observation and statistics. Data are shown as mean ± s.d. (**f**) Knockdown efficiency of siRNA targeting Bub3. (**g**) The co-IP experiment detecting the interaction between NKAP and Bub3. Flag-NKAP and His-Bub3 were transfected into HEK293T cells. Flag-NKAP IPs were immunoblotted with indicated antibodies. (**h**) NKAP directly binds Bub3. In vitro translated Bub3 was incubated with GST, GST-NKAP. The bound proteins were subjected to SDS-PAGE followed by immunoblot. The amounts of recombinant proteins from the same reaction were analysed by SDS-PAGE and Coomassie blue staining. Arrowheads indicate the positions of corresponding proteins. (**i**) Western blot analysis for the expressions of indicated siRNA-resistant NKAP deletion mutants in cells as described in Fig. 6e.

Supplementary Figure 8

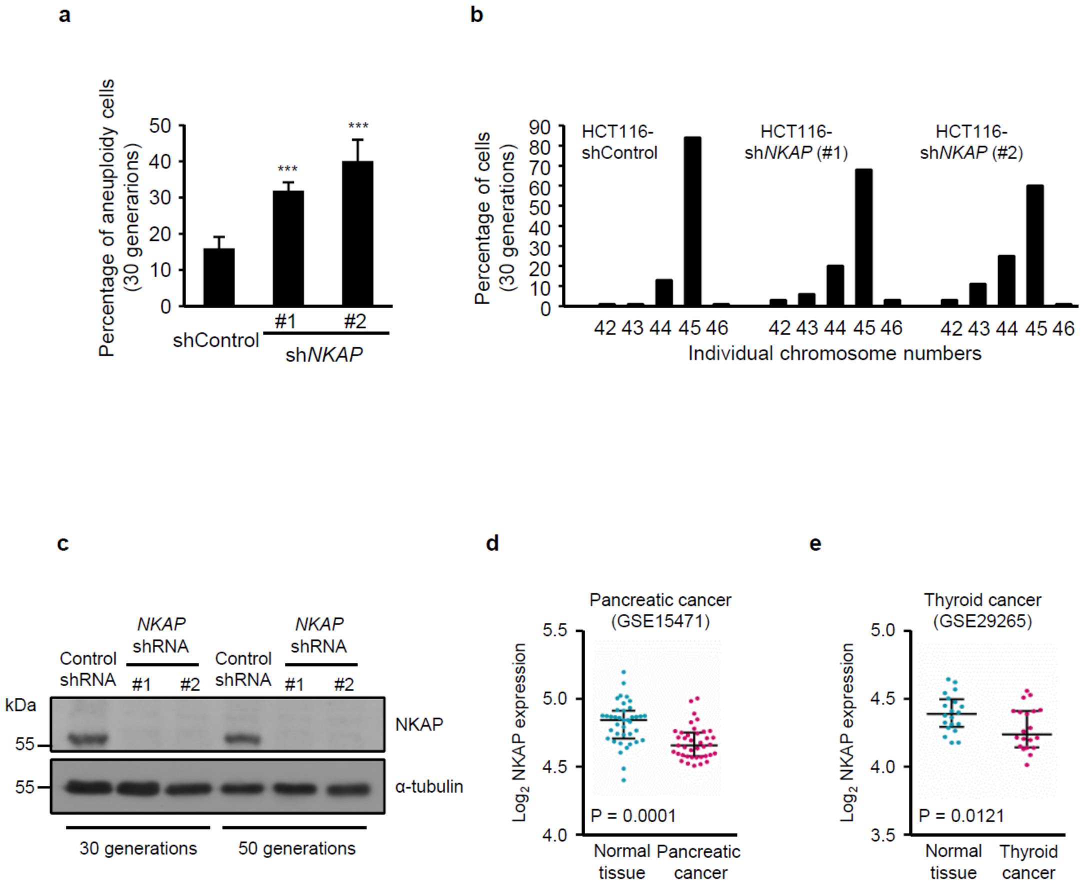

Supplementary Figure 8 NKAP deficiency leads to aneuploidy and is observed in human cancers. (**a**) HCT116 cells were stably infected with lentivirus expressing control or *NKAP* shRNAs. The proportion of cells with a karyotype deviating from the modal chromosome number was determined within 30 generations in cells as indicated in **b** (n > 100 cells). Data are representative of three independent experiments and shown as mean ± s.d. *** P< 0.001 (One-way ANOVA). (**b**) Individual chromosome numbers from metaphase spreads of cultured HCT116 and HCT116-*NKAP* shRNA cells were analysed within 30 generations. (n > 100 cells). Cells were randomly selected for observation and statistics. (**c**) Western blot analysis for NKAP protein level in cells as described in **a** and Fig. 7a. (**d**) Decreased NKAP expression in pancreatic cancer. Each circle represents an individual sample of

human pancreatic cancer (*n* = 39 for normal tissue; *n* = 39 for pancreatic cancer). *P* = 0.0001 (Two-sided Mann–Whitney U test). (**e**) Reduced NKAP expression in thyroid cancer. Each circle represents an individual sample of human thyroid cancer (*n* = 20 for normal tissue; *n* = 20 for thyroid cancer). *P* = 0.0121 (Two-sided Mann–Whitney U test).

Supplementary Figure 9

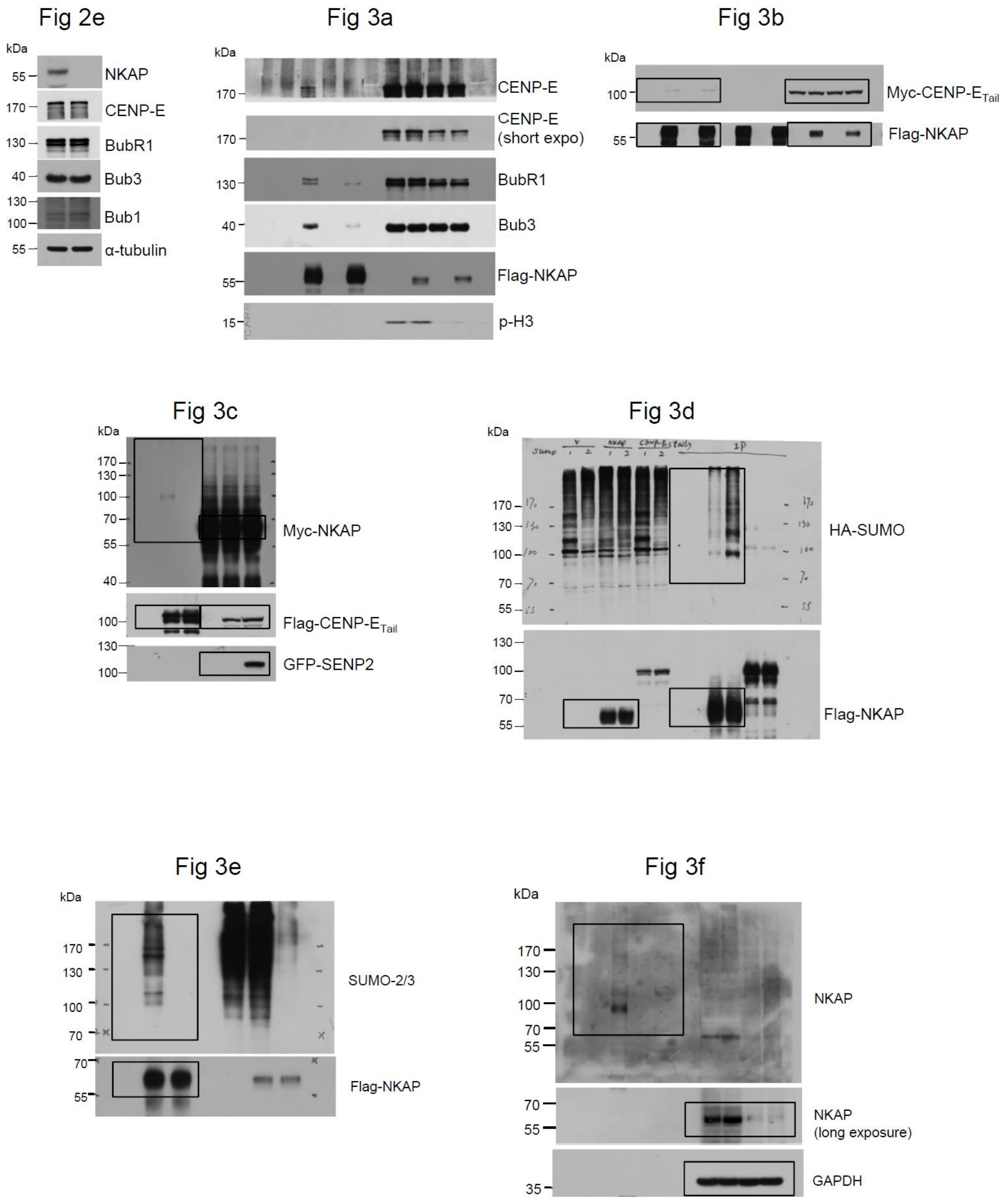

Figure S9. continued

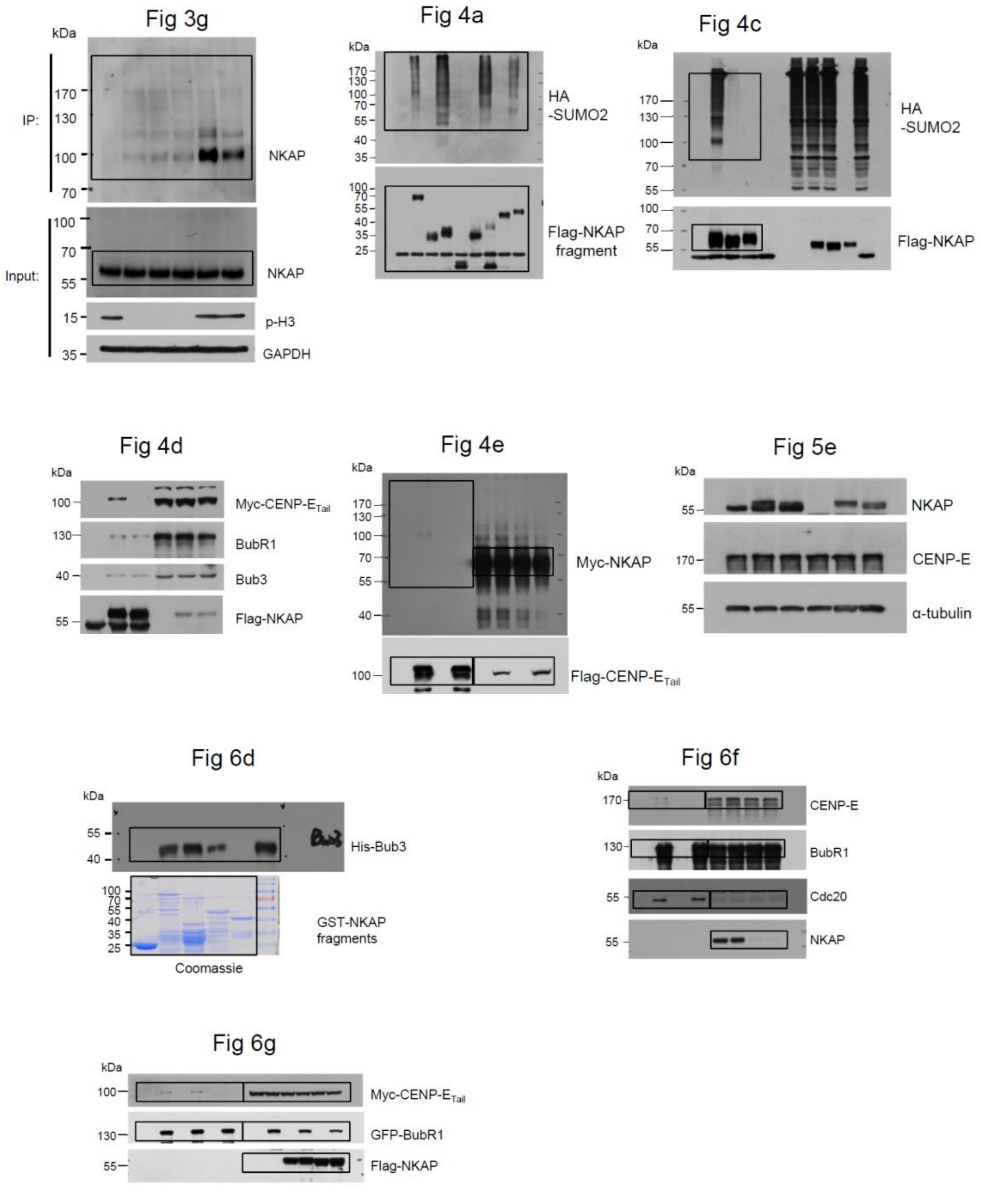

Supplementary Figure 9. Images of uncropped scans of representative immunoblots and Coomassie blue staining gel. Black boxes indicate the cropped regions that are shown in the indicated figures.

Supplementary Table 1. Information of the NKAP expression plasmids

Plasmid	Vector	Primer	Restriction Sites
Flag-NKAP (WT)-F	pcDNA3.0-Flag	5' CGCGGATCCATGGCTCCGGTGTCCGGCT 3'	BamHI/XhoI
Flag-NKAP (WT)-R	pcDNA3.0-Flag	5' CCGCTCGAGTTATTTGTCATCCTTCCCTTTGGT 3'	BamHI/XhoI
Flag-NKAP (1-207)-F	pcDNA3.0-Flag	5' CGCGGATCCATGGCTCCGGTGTCCGGCT 3'	BamHI/XhoI
Flag-NKAP (1-207)-R	pcDNA3.0-Flag	5' CCGCTCGAGTTACTTCTTATGTTTTCTTTTCGATG 3'	BamHI/XhoI
Flag-NKAP (208-415)-F	pcDNA3.0-Flag	5' CGCGGATCCATGTATTCTGAAGATAGCGACAGTG 3'	BamHI/XhoI
Flag-NKAP (208-415)-R	pcDNA3.0-Flag	5' CCGCTCGAGTTATTTGTCATCCTTCCCTTTGGT 3'	BamHI/XhoI
Flag-NKAP (1-138)-F	pcDNA3.0-Flag	5' CGCGGATCCATGGCTCCGGTGTCCGGCT 3'	BamHI/XhoI
Flag-NKAP (1-138)-R	pcDNA3.0-Flag	5' CCGCTCGAGTTATCCAATTCTCTCTCTCTCACTT 3'	BamHI/XhoI
Flag-NKAP (139-276)-F	pcDNA3.0-Flag	5' CGCGGATCCATGGAATTGGGAGCTCCTGAAGTA 3'	BamHI/XhoI
Flag-NKAP (139-276)-R	pcDNA3.0-Flag	5' CCGCTCGAGTTAGGGATTTTCCAGAAACTCTTCT 3'	BamHI/XhoI
Flag-NKAP (277-415)-F	pcDNA3.0-Flag	5' CGCGGATCCATGTGGAAGGATCGAACAAAGGCT 3'	BamHI/XhoI
Flag-NKAP (277-415)-R	pcDNA3.0-Flag	5' CCGCTCGAGTTATTTGTCATCCTTCCCTTTGGT 3'	BamHI/XhoI
Flag-NKAP (1-276)-F	pcDNA3.0-Flag	5' CGCGGATCCATGGCTCCGGTGTCCGGCT 3'	BamHI/XhoI
Flag-NKAP (1-276)-R	pcDNA3.0-Flag	5' CCGCTCGAGTTAGGGATTTTCCAGAAACTCTTCT 3'	BamHI/XhoI
Flag-NKAP (139-415)-F	pcDNA3.0-Flag	5'CGCGGATCCATGGAATTGGGAGCTCCTGAAGTA 3'	BamHI/XhoI
Flag-NKAP (139-415)-R	pcDNA3.0-Flag	5' CCGCTCGAGTTATTTGTCATCCTTCCCTTTGGT 3'	BamHI/XhoI
GFP-NKAP (res-WT)-F	pEGFP-N1	5' CCGGAATTCGCCACCATGGCTCCGGTGTCCGGCT 3'	BamHI/EcoRI
GFP-NKAP (res-WT)-R	pEGFP-N1	5' CGCGGATCCCGTTTGTCATCCTTCCCTTTGGT 3'	BamHI/EcoRI
GFP-NKAP (res-14KR)-F	pEGFP-N1	5' CCGGAATTCGCCACCATGGCTCCGGTGTCCGGCT 3'	BamHI/EcoRI
GFP-NKAP (res-14KR)-R	pEGFP-N1	5' CGCGGATCCCGTTTGTCATCCTTCCCTTTGGT 3'	BamHI/EcoRI
RFP-NKAP (res-WT)-F	pIRES2-DsRed	5' CCGGAATTCGCCACCATGGCTCCGGTGTCCGGCT 3'	BamHI/EcoRI
RFP-NKAP (res-WT)-R	pIRES2-DsRed	5' CGCGGATCCCGTTTGTCATCCTTCCCTTTGGT 3'	BamHI/EcoRI
RFP-NKAP (res-14KR)-F	pIRES2-DsRed	5' CCGGAATTCGCCACCATGGCTCCGGTGTCCGGCT 3'	BamHI/EcoRI
RFP-NKAP (res-14KR)-R	pIRES2-DsRed	5' CGCGGATCCCGTTTGTCATCCTTCCCTTTGGT 3'	BamHI/EcoRI
Myc-CENP-E(tail)-F	pXJ40-Myc	5' CGCGGATCCAAGGATTTAGATAAAT 3'	BamHI/EcoRI
Myc-CENP-E(tail)-R	pXJ40-Myc	5' CGGGGTACCCTACTGAGTTTTGCACTCAG 3'	BamHI/EcoRI

Supplementary Table 2. Information of antibodies

Name	Source	Host organism	WB/IF/IP
Cdc20	Santa Cruz (sc-13162)	mouse, mono	WB
Normal mouse IgG	Santa Cruz (sc-2025)	mouse, mono	IP
Normal rabbit IgG	Santa Cruz (sc-2027)	rabbit, poly	IP
Myc	Santa Cruz (sc-789)	rabbit, poly	WB
GFP	Santa Cruz (sc-9996)	mouse, mono	WB
CENP-F	Santa Cruz (sc-22791)	rabbit, poly	WB/IF
CENP-E	Santa Cruz (sc-22790)	rabbit, poly	IF
CENP-E	Abcam (ab5093)	mouse, mono	IF
HA	Santa Cruz (sc-805)	rabbit, poly	WB
KNL1	Novus (NBP1-42704)	rabbit, poly	IF
Mis12	Santa Cruz (sc-98368)	rabbit, poly	IF
MCAK	Cytoskeleton (AKIN05)	rabbit, poly	IF
dynein HC	Proteintech (12345-1-AP)	rabbit, poly	IF
Hec1	Abcam (ab3613)	mouse, mono	WB/IF
Bub3	Abcam (ab131157)	rabbit, poly	WB
Bub1	Abcam (ab54893)	mouse, mono	WB/IF
NKAP	Abgent (AP12401b)	rabbit, poly	WB
NKAP	Self-made	mouse, mono	IF
Flag	Sigma (F3165)	mouse, mono	WB/IF
α-tubulin	Sigma (t5168)	mouse, mono	WB/IF
Mad2	BD (610678)	mouse, mono	WB
Histone H4	Abcam (ab7311)	rabbit, poly	WB
His	MBL international (D291-3)	mouse, mono	WB
RFP	MBL international (PM005)	rabbit, poly	WB
GST	MBL international (M071-3)	mouse, mono	WB
BubR1	Bethyl Laboratories (A300-386A)	rabbit, poly	WB/IF/IP
Aurora B	Bethyl Laboratories (A300-431A)	rabbit, poly	IF
phospho-histone H3 (Ser 10)	Millipore (05-817R)	rabbit, poly	WB
Nuf2	GeneTex Inc. (GTX110734)	rabbit, poly	IF
CREST	Antibodies Inc. (15-234-0001)	human,antisera	IF
CLIP-170	Novartis (NBP1-85568)	rabbit, poly	IF
CENP-E (HpX)	Gift from Dr. Don Cleveland, University of California, San Diego	mouse, mono	WB
Ska1	Gift from Hongtao Yu, (UTSouthwestern), Dallas, Texas	mouse, mono	IF
Mad1	Gift from Hongtao Yu, (UTSouthwestern), Dallas, Texas	rabbit, poly	IF

Supplementary Methods：

Plasmids construction: To create siRNA resistant NKAP plasmid, the sequence (ACAAGTGAAGAAATTGCA) in NKAP corresponding to the siRNA was changed to (ACcAGcGAgGAgATcGCg) without changing the encoded amino acids.

NKAP (14KR) clones were constructed using two rounds of PCR. 14KR mutated fragment (832-1248bp) was synthesized by Integrated DNA Technologies Corporation. Four primers were used: A-F (5'CGCGGATCCATGGCTCCGGTGTCCGGCT3'); B-R (5'CCGCTCGAGTTACCTGTCATCCTTCCCTTTGGT3'); B-F (agtttctggaaaatcccTGG AGGGATCGAACAAA) and A-R (tttgttcgatccctccaGGGATTTTCCAGAAACT). In the first round of PCR, primers A-F and A-R were used to obtain the PCR product A (1-848bp of NKAP mRNA), and primers B-F and B-R were used to obtain the PCR product B (815-1248 of NKAP mRNA). In the second round PCR, product A and B were mixed (1:1 ratio) and then used as template. Product A and B contain the same 34bp nucleotides (consisted of 815-848bp of NKAP mRNA) and they act as template for each other. After 5 cycles for PCR, primers A-F and B-R were added to expand the NKAP (14KR) cDNA sequence and to add restriction sites on both sides of the NKAP (14KR) cDNA sequence. The PCR sample of the second round PCR was used for cloning.

NKAP inserts were subcloned into pcDNA3.0-Flag, pXJ40-Myc, pIRES2-DsRed, pEGFP-N1 and pGEX-4T-1 vectors.

Supplementary References：

1. Neumann B*, et al.* Phenotypic profiling of the human genome by time-lapse microscopy reveals cell division genes. *Nature* **464**, 721-727 (2010).
2. Merbl Y, Refour P, Patel H, Springer M, Kirschner MW. Profiling of ubiquitin-like modifications reveals features of mitotic control. *Cell* **152**, 1160-1172 (2013).
3. Olsen JV*, et al.* Quantitative phosphoproteomics reveals widespread full phosphorylation site occupancy during mitosis. *Sci. Signal.* **3**, ra3 (2010).
